# Persistence of human enteric viruses in artificial and human saliva

**DOI:** 10.1371/journal.pone.0339724

**Published:** 2025-12-26

**Authors:** Sharon C. Kosgei, Olivia N. Birch, Justin C. Greaves

**Affiliations:** Department of Environmental and Occupational Health, School of Public Health, Indiana University-Bloomington; Instituto Butantan, BRAZIL

## Abstract

Enteric viruses, such as Adenovirus 41 (AdV41) and Coxsackievirus B3 (CVB3), are significant contributors to gastrointestinal infections, particularly among young children and immunocompromised individuals. While fecal-oral transmission is the primary route of infection, emerging evidence indicates that saliva may also function as a reservoir for these viruses, posing a potential risk for oral transmission. Previous studies have primarily focused on the decay of viruses in aqueous matrices, like wastewater and freshwater, but the persistence of these viruses in human saliva remains underexplored. This study aimed to investigate the persistence of AdV41 and CVB3 in both human and artificial saliva under various conditions, including the presence of fecal particles and specific oral bacteria. Our findings demonstrated that human saliva significantly reduced viral stability compared to artificial saliva, with marked reductions in viral titers observed within 24–48 hours. Particularly, the presence of fecal particles in both saliva types extended CVB3 viral persistence, suggesting a protective effect due to particle adsorption. However, AdV41 demonstrated an opposite trend when in the presence of fecal particles, suggesting virus-specific differences in how particulate matter influences stability. Additionally, specific oral bacteria, such as *Streptococcus mutans*, significantly enhanced CVB3 stability, with a mean viral recovery of 23.2% of the viral titer after 24 hours in the presence of the bacteria compared to 0.7% in their absence (*p* = 0.001). This study shows complex interactions between viruses, oral bacteria, and fecal particles within the oral environment, emphasizing the need for further research on oral viral persistence and transmission dynamics. Understanding these mechanisms behind viral persistence in saliva can inform public health strategies aimed at mitigating the risk of transmission.

## 1.0 Introduction

The oral cavity is one of the first points of contact with various external components. Because of this, the oral cavity has been identified as a diverse ecosystem harboring various microorganisms including viruses, fungi, and bacteria which interact with one another and the hosts immune defenses [1–4]. Saliva, secreted by the salivary glands in the oral cavity, is an intricate combination of electrolytes, proteins, enzymes, fluids, and macromolecules that maintain oral homeostasis [5,6]. It contains antimicrobial and immune components such as defensins, histatins, lysozyme, lipocalin, mucins, enzymes like amylase that work collectively to inhibit dangerous external pathogens by exerting selective pressure on microbial populations [5,7]. Despite the antimicrobial properties of saliva, enteric viruses, such as CVB3 and AdV41, can persist through the oral cavity and infect the latter parts of the gastrointestinal system.

Enteric viruses are a significant cause of morbidity and mortality worldwide, particularly among children under five [8]. Among these pathogens, AdV41 and CVB3 pose major public health concerns [9,10]. AdV41 is a double stranded DNA virus that is strongly associated with severe gastrointestinal illness in children, contributing to an estimated 52,613 deaths globally among those under five [10–12]. In contrast, Coxsackieviruses, are single stranded RNA viruses most linked to severe infections in neonates and infants under one year old [13,14]. It is known to cause over 40,000 infections annually in the United states [9] including myocarditis [15], and aseptic Meningitis [16].These enteric viruses primarily affect the lower gastrointestinal tract [8,9,17]. However, emerging evidence suggests that their presence is not confined to this part of the gut as studies have highly detected these viruses in the saliva of infected individuals with a distinct 30-day persistence period, indicating a potential alternative route of transmission [18,19].

The detection of enteric viruses in saliva has gained attention in recent years as it is minimally invasive and easy to implement in any setting [20,21]. However, it remains unclear how long viruses, such as AdV41 and CVB3, can survive in saliva. Furthermore, recent studies have observed enteric viruses, such as AdV41 and CVB3, to interact with fecal particles and bacteria allowing for the extension of their survival [22–24]. Viruses can adsorb on particulate matter which shield them against proteolytic enzymes, oxidative damage, ultraviolet radiation, and virus aggregation formation therefore reinforcing viral capsid integrity [24]. There is potential for this protective effect of fecal particles to be replicated in the oral cavity where fecal particles protect viruses from saliva [25,26]. Viruses are also able to bind to various components of bacteria which allow them to remain stable against the various environmental factors that might degrade them [18,27,28]. As the oral cavity is composed of a diverse bacterial community of over 770 bacterial species, the presence of oral bacteria may further influence viral persistence similar to what is found in the lower gastrointestinal track [29].

Despite these findings, the ability of enteric viruses to persist in human saliva has been underexplored. Furthermore, the interaction of oral bacteria and fecal particles with viruses has not been well characterized. Most viral persistence studies have focused on aqueous matrices such as wastewater [30,31], fresh waters [32], and marine waters [33,34], neglecting biological fluids such as saliva. Additionally, while artificial saliva has been used in biological experiments related to medical, dental and toxicology studies, its formulations in modeling human saliva is questionable as there is no universal artificial saliva model [35].

The current study aimed to address this major research gap by evaluating the persistence of the two enteric viruses, AdV41 and CVB3, across multiple matrices including human saliva and artificial saliva at different time points. Our study also examined the role of oral bacteria and fecal particles in extending viral persistence. Investigating these interactions between bacteria, fecal particulates, and viruses will contribute to the understanding of the transmission pathways in humans and improve future assessments of risk of infection from these types of viruses.

## 2.0 Materials and methods

### Cell culture

HeLa (ATCC CCL-2) and HEK 293 (ATCC CRL-1573) cell lines were cultured and maintained under standard conditions according to manufacturer’s instructions [36]. HeLa cells were grown in Dulbecco’s Modified Eagle Medium (DMEM, ATCC 30–2002), while HEK 293 cells were maintained in Eagle’s Minimum Essential Medium (EMEM, ATCC 30–2003). Both media were supplemented with 10% fetal bovine serum (FBS) and 1% antibiotics. Cells were incubated at 37°C and 5% CO₂. Upon 100% confluency, HeLa cells were seeded in six well plates while HEK 293 were seeded in T25 flasks for subsequent experiments.

### Virus strains and sample sources

Human adenovirus type 41 (ATCC® VR-930™) and human coxsackievirus B3, Nancy strain (ATCC® VR-30™) was obtained from the American Type Culture Collection (ATCC, Manassas, VA, USA). AdV41 and CVB3 were propagated in HEK293 and HeLa cells, respectively. Pooled human saliva was purchased from Innovative Research Inc. (Novi, MI, USA). This product retains its native biochemical constituents, including electrolytes, mucins, enzymes (e.g., α-amylase, peroxidase, and lysozyme), antimicrobial peptides, lactoferrin, and immunoglobulins (IgA, IgG, IgM). Artificial saliva was obtained from Pickering Laboratories Inc. (Mountain View, CA, USA, Cat. No. 1700−0317, 1 × 200 mL). The artificial saliva formulation conformed to ASTM standards E2720-16 and E2721-16, included mucin as a key component, minerals (potassium chloride, sodium chloride, calcium chloride, potassium dihydrogen phosphate), and a pH formulation of 7.0 stabilized for experimental use. This formulation was selected for its chemical stability and close physiological similarity to human saliva. Pooled human saliva was stored at −20°C and artificial saliva at 4°C as per manufacturer’s instructions until use. Fecal samples negative for enteric viruses were obtained from Indiana University Health Indianapolis Hospital and stored at −80°C until use.

### AdV41 virus propagation and quantification

The decay kinetics of AdV41 were assessed under five different matrix conditions to evaluate viral persistence. The matrices included: Phosphate-buffered saline (PBS) containing virus only, human saliva with virus, human saliva with fecal particles and virus, artificial saliva with virus, and artificial saliva with fecal particles and virus. Each matrix type had 400 μL, of the respective solution spiked with 4 μL of 10^5^ Infectious units (IU) of AdV41 to ensure quantifiable viral detection over the experimental period, consistent with concentrations reported in environmental wastewater 10^8^ genome copies per liter (GC)/L, treated effluent 10⁰ to 10⁷ GC/L, and clinical fecal matrices 10^8–^10^11^ genome copies per gram (GC/g) [12,37]. PBS served as the baseline control, while saliva matrices represented biologically active environments relevant to oral exposure. A PBS with fecal particle condition was excluded because it lacks physiological relevance. Fecal samples were collected using a sterile thin spatula, and approximately 0.1 g of material was aliquoted and rehydrated in 400 μL of either human or artificial saliva prior to viral spiking. Each rehydrated matrix was inoculated with 4 μL of AdV41 stock suspension and incubated at 37°C for the designated time points (0, 2, 5, 24, 72, and 504 hours). At each interval, viral particles were purified by adding 10% (v/v) chloroform, vortexed followed by centrifugation at 13,000 rpm for 10 minutes. The resulting supernatant was carefully collected for subsequent viral quantification.

Integrated cell culture polymerase chain reaction (ICC-PCR) method was utilized to quantify AdV41 as described previously by Rodriguez et al. [38]. HEK293 cells in T25 flasks were infected with each sample type diluted in no serum media to a volume of 1 mL and incubated with a 1-hour adsorption time. After 1 hour, cells were supplemented with Eagle’s Minimum Essential Medium with 2% fetal bovine serum media and incubated for 3 days at 37^◦^C and 5% CO₂. Triplicate infection experiments were done at different time points 0, 2, 5, 24, 72, and 504-hours post-inoculation.

After the incubation, the media was discarded and cells eluted from the flask using lysis buffer (Buffer 166 RLT) from the RNeasy® kit (QIAGEN, Hilden, Germany). Homogenization of lysates was done using the QIAshredder kit and total RNA extracted using the QIAGEN ®RNeasy ®Micro Kit (Hilden, Germany) following manufacturer’s instructions including an in-column DNase treatment to eliminate DNA.

Reverse transcription of mRNAs to cDNAs was performed using Oligo dT_18_ primer (Thermo Fisher Scientific, Waltham, MA, USA) and M-MLV reverse transcriptase (Invitrogen, Carlsbad, CA, USA). Briefly, 4 μL of mix 1 (containing 2.5μM of 50μm oligo dT_18_, 0.5mM of 10mM dNTP) was added to 8 μL of RNA sample, heated at 65°C for 5 minutes, and cooled to 4°C on thermocycler. 8 μL of mix 2 comprising of 5X first strand buffer, 10mM of 100mM 0.1MDTT solution, 80 μL of RNAse inhibitor (40 units/mL) and 200 μL of RT enzyme (200 units/mL) (Invitrogen, Carlsbad, CA) was added to the sample and incubated at 37°C for 50 mins, 70 ^◦^C for 15 mins and cooled to 4°C. Real time polymerase chain reaction (qPCR) was performed to quantify viral cDNA using primers and cycling conditions described by Greaves et al. [39]. The amplification protocol began with an initial enzyme activation step at 95 °C for 15 minutes, followed by 40 cycles of denaturation at 95°C for 10 seconds and annealing at 60°C for 45 seconds. Each 20 µL reaction contained 10 µL of 2 × QuantiTect Probe Master Mix (Qiagen, Hilden, Germany), 2 µL of a 10 × primer-probe mix providing final concentrations of 500 nM for each primer and 250 nM for the probe, and 6 µL of RNase-free water. Subsequently, 2 µL of extracted viral DNA was added to the reaction mixture.

Quantification was based on a five-point standard curve generated from serial dilutions (10⁰-10 ⁻ ⁸) of propagated AdV41 viral stock. The curve established a linear relationship between cycle threshold (Cq) values and genome copy number. Each qPCR run included triplicate standards, triplicate test samples, and a no-template control to monitor for contamination. Multiple calibration runs were conducted, and average values derived from the standard curve were as follows: R^2^ = 0.971, slope = −3.483, y-intercept = 46.389, and efficiency = 0.59. These parameters indicate consistent assay performance across runs and an acceptable level of quantitative accuracy for environmental samples.

### CVB3 sample preparation and quantification

Multiple experimental replicates were performed using different sample matrices including PBS, human saliva, human saliva with fecal particles, artificial saliva and artificial saliva with fecal particles in 400 μL volumes inoculated with 10^5^ PFU of CVB3 a concentration chosen to reflect environmentally relevant levels of 10³-10⁵ GC/L typically detected in wastewater, as well as viral loads shed in human stool during acute infection, which range from approximately 10⁵-10⁹ GC/g [40,41]. Samples were incubated at 37^◦^C prepared for different time points including 0, 2, 5, 24, and 48 hrs. Following incubation, each sample was purified with 10%(v/v) chloroform, and centrifuged at 13,000RPM for 10 mins. The resulting supernatant was collected and serially diluted up to 10 ⁻ ^5^ for plaque quantification [42].

To evaluate impact of oral bacteria on stability of CVB3, additional samples were prepared using bacterial strains *Bacillus subtilis* (common gut bacteria), *Streptococcus mutans* (common oral bacteria), and *Streptococcus Downei* (common oral bacteria) [43,44]. The bacterial strains were obtained from ATCC and cultured in the appropriate broth for 48 hours under standard aerobic conditions to reach a concentration of 10^6^ CFU/mL. Additionally, a mixed population of salivary bacteria were established by culturing pooled human saliva in Brain Heart Infusion (BHI) broth for 48 hours.

Following incubation, 10^6^ CFU of the respective bacterial culture was mixed with 10^5^ PFU of CVB3 in pooled human saliva to approximate the natural oral bacterial load (10^6^–10^9^ CFU/mL) and to maintain physiologically relevant virus-to-bacteria ratios [45]. The resulting mixtures were treated with 10% (v/v) chloroform and centrifuged to purify the viral particles. At the 24-hour post-incubation time point, they were assessed for viral stability.

CVB3 was quantified using plaque assay as previously described [43]. Briefly, HeLa cells were inoculated with the various samples containing CVB3 at different time points then samples removed after 1–2 hours post incubation. Agar Overlay (½ agarose/ ½ MEM 2x (2% Antibiotics, 4% Fetal Bovine Serum) was added and incubated for 2 days. Agar was removed and BD BBL™ Gram Stain Crystal Violet reagent (Becton, Dickinson and Company, Sparks, MD, USA) used to visualize plaques.

### Statistical analysis

The data analysis was conducted using ANOVA on GraphPad Prism v10 (Boston, MA, USA), with independent factors being considered. Graphs and figures were made using GraphPad Prism v 10 (Boston, MA, USA). The decay rate constants (k) were calculated using built-in nonlinear regression tools in GraphPad Prism, and goodness of fit was assessed via R² values. Half-lives t_½_ = ln (2)/k were also estimated for comparison across conditions.

**For calculating the rate of decay, the decay curve was fit to the following formula:**


$$C_{T}=C_{\text{0}}e^{-kt}$$

In this equation, C_T_ represents the concentration of the pathogen at time *T*, *C*_*0*_ represents the initial concentration of the pathogen at time 0, and *k* is representative of the decay rate. Comparison between decay rates were done using one way ANOVA with multiple comparisons.

## 3.0 Results

### Saliva influences stability of enteric viruses CVB3 and AdV41

**Fig 1:**
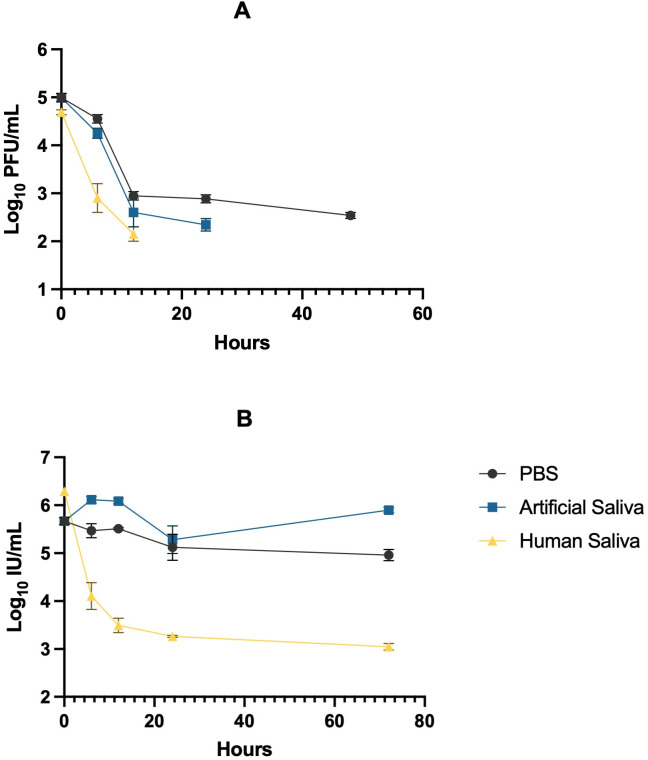
Enteric viral decay kinetics in PBS, artificial saliva, and human saliva at 37 °C. Viral titers were quantified and expressed as log₁₀ PFU/mL for CVB3 (panel a) and log₁₀ IU/mL for AdV41 (panel b). Panel (a) shows CVB3 infectivity over a 48-h incubation, while panel (b) presents AdV41 persistence over 72 h. Data points represent means ± SD of biological triplicates (n = 3). All time points were tested, however, values below the detection limit were not plotted on the graph.

To assess the stability of CVB3 in saliva, we investigated viral decay kinetics in PBS, artificial saliva, and human saliva over a 48-hour period at 37°C. Viral titers were quantified by plaque assay and expressed as log₁₀ PFU/mL (Fig 1a). At time zero hours post incubation, the viral titer across all matrices were similar averaging between 4.6–5.0 log₁₀ PFU/mL showing equal input across sample conditions. There was no significant difference in CVB3 concentrations between PBS and artificial saliva (*p* = 0.2085), however, significant differences were observed between PBS and human saliva, as well as between artificial saliva and human saliva (*p* < 0.0001; S2 Table). After 2 hours of incubation, viral titers declined across all matrices, with a gradual decrease in PBS and artificial saliva (*p* < 0.0001; S2 Table). CVB3 infectivity in human saliva declined rapidly, reaching undetectable levels by 5 hours. Although all-time points up to 48 hours were tested, viral concentrations in human saliva remained below the detection limit beyond 5 hours. Values below the detection limit were excluded from statistical analyses and are not shown in the plotted data. At this point, viral titers in human saliva were significantly lower than those in PBS (*p* < 0.0001; S2 Table) and artificial saliva (*p* = 0.0063; S2 Table). By 24 hours, the average titer in PBS remained above 2.90 log₁₀ PFU/mL, and in artificial saliva it was 2.35 log₁₀ PFU/mL, indicating sustained infectivity under these conditions (*p* < 0.0001; S2 Table). In contrast, CVB3 concentrations decreased more rapidly in human saliva, with the virus becoming undetectable in most replicates by 24 and 48 hours. Overall, significant reductions in CVB3 concentrations were evident between human saliva and both PBS and artificial saliva matrices (*p* < 0.0001; S2 Table). These data suggests that CVB3 was more stable in PBS and artificial saliva compared to human saliva.

To assess the stability of AdV41, we evaluated viral decay kinetics in PBS, artificial saliva, and human saliva over a 72-hour incubation period at 37 °C. Viral titers were quantified by ICC-PCR and expressed as log₁₀ IU/mL (Fig 1b). AdV41 concentrations remained relatively stable in PBS and artificial saliva throughout the incubation, maintaining titers of 4.96 log₁₀ IU/mL and 5.90 log₁₀ IU/mL at 72 hours, respectively. No significant differences were observed between PBS and artificial saliva at early time points (*p* > 0.9999), between PBS and human saliva and artificial and human saliva at time zero (*p* = 0.2120; S3 Table) respectively. However, artificial saliva and human saliva exhibited significantly different viral concentrations across all the other time points (*p* < 0.0001; S3 Table). Beginning at 2 hours, viral titers in human saliva declined sharply compared to both PBS and artificial saliva (*p* < 0.0001; S3 Table), with concentrations reaching 3.05 log₁₀ IU/mL by 72 hours. Interestingly, there was a significant difference between PBS and artificial saliva at 72 hours (*p* = 0.0062; S3 Table), suggesting that the organic and ionic components of artificial saliva, such as mucins and electrolytes, may provide a stabilizing microenvironment that enhances AdV41 persistence relative to PBS. Collectively, these findings indicate that while human saliva promotes rapid viral decay and, artificial saliva supports prolonged AdV41 stability compared to PBS.

### Fecal particles essential for CVB3 stability in saliva

**Fig 2:**
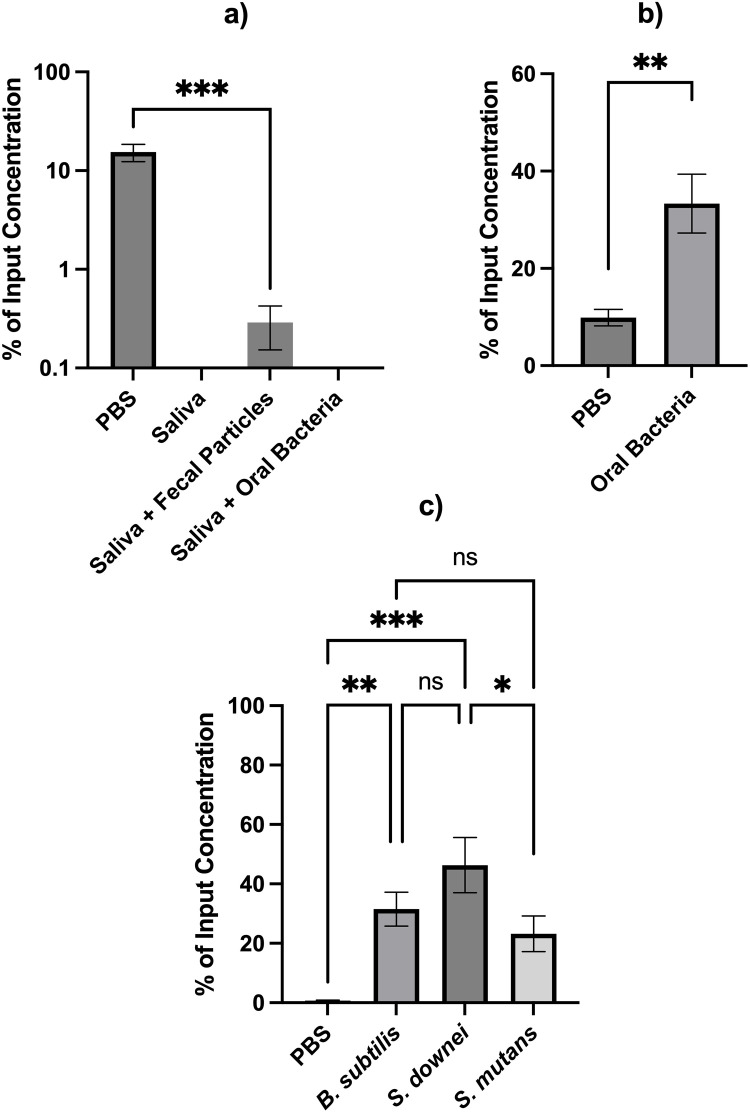
Oral bacteria and fecal particles can extend CVB3 infectivity. Panel a shows incubation of CVB3 in PBS, in human saliva, in human saliva with fecal particles, or in human saliva with oral bacteria at 37°C for 24 hours represented as % of input concentration. Panel b shows incubation of CVB3 in PBS in the presence and absence of multiculture oral bacteria grown in BHI represented as % of input concentration. Panel c shows incubation of CVB3 in PBS, *B. subtilis, S. downei*, and *S. mutans* represented as % of input concentration with statistical significance represented as ns (not significant), *p < 0.05, **p < 0.01, and ***p < 0.001(mean ± SD, n = 3). All time points were tested, however, values below the detection limit were not plotted on the graph.

The presence of particulate matter has been shown in previous studies to enhance viral persistence in aqueous environments. To evaluate its influence on CVB3 stability and compare it with the effect of oral bacteria, we incubated CVB3 for 24 hours at 37 °C in four matrices: PBS, human saliva, human saliva containing fecal particles, and human saliva supplemented with oral bacteria (Fig 2a). A significant reduction in viral titers was observed in all saliva-based matrices compared to PBS (*p* = 0.0003–0.0006; S8 Table). CVB3 in PBS retained an average of 15.4% of the input viral titer, whereas incubation in human saliva or saliva containing oral bacteria resulted in near-complete loss of infectivity, with virus levels falling below detectable limits with no significant difference in the two saliva matrices (*p* > 0.9999; S8 Table). In contrast, CVB3 incubated in saliva containing fecal particles showed detectable, and significantly reduced, concentrations corresponding to 0.29% of the input titer. Although still markedly lower than PBS (*p* = 0.0006; S8 Table), this finding suggests that fecal particles exert a modest stabilizing effect on CVB3 compared to oral bacteria, likely by providing microenvironments or adsorptive surfaces that mitigate viral inactivation in saliva.

Prior studies show that the presence of various bacteria enhances viral infectivity and stability. Therefore, we determined whether oral bacteria could influence CVB3 stability. CVB3 was incubated for 24 hours at 37°C in PBS alone or PBS supplemented with saliva sourced oral bacteria grown in BHI. Viral titers were measured and expressed as a percentage of input virus. (Fig 2b). A significant difference was observed (*p* < 0.01) in the absence of oral bacteria (PBS alone) compared to the presence of oral bacteria. CVB3 was significantly higher in the presence of oral bacteria compared to the absence of oral bacteria after 24 hours.

We further incubated 10^5^ plaque-forming units (PFU) of CVB3 with 10^6^ PFU of either *Bacillus subtilis, Streptococcus mutans,* and *Streptococcus downei* in PBS at 37°C for 24 hours (Fig 2c). We found that incubation with bacteria allowed for concentrations of CVB3 to be significantly higher compared to being in PBS alone. Notably, *S. downei* exhibited the greatest stabilizing effect, with a mean viral recovery of 46.32% of the initial viral titer (*p* = 0.001; S9 Table). *B. subtilis* also enhanced viral stability, showing a mean recovery of 31.52% (*p* = 0.0098; S9 Table). Similarly, *S. mutans* promoted viral stability, yielding a mean recovery of 13.9%, which approached statistical significance (*p* = 0.051; S9 Table). These findings indicate that individual oral bacteria particularly *S. downei* and *S. mutans* can enhance CVB3 infectivity in a manner comparable to intestinal bacteria such as *B. subtilis*.

### Fecal particles enhance viral stability of CVB3 in human and artificial saliva

**Fig 3:**
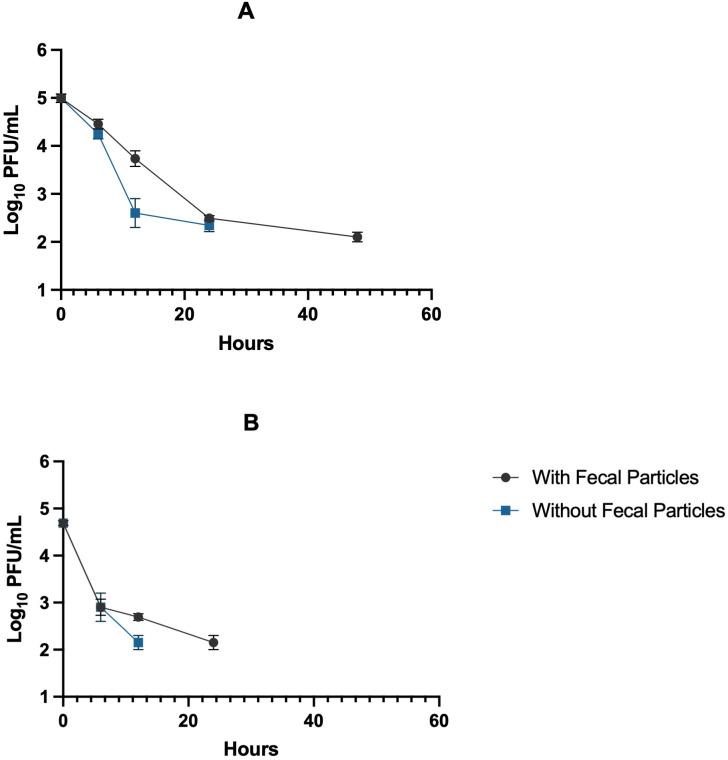
Decay of CVB3 in artificial saliva (A) and human saliva (B). CVB3 was incubated at 37 °C for 48 hours in the presence and absence of fecal particles represented as log₁₀ PFU/ mL (mean ± SD, n = 3). All time points were tested, however, values below the detection limit were not plotted on the graph.

We then incubated CVB3 in artificial saliva either with or without fecal particles at 37 °C. Viral titers were then measured at 0, 2, 5, 24, and 48 hours by plaque essay (Fig 3a). CVB3 decay was delayed in artificial saliva with fecal particles with titers averaging 2.10 log₁₀ PFU/mL by 48 hours compared to artificial saliva without fecal particles whose viral titer averaged at 2.35 log₁₀ PFU/mL and no detection by 48 hours compared to the initial 0 time point (*p* < 0.0001; S4 Table). Similarly, CVB3 incubated in saliva containing fecal particles demonstrated a slower decline, with significant differences emerging between 2 and 24 hours (*p* = 0.0035; S4 Table) and between 2-hour saliva without fecal particles and 24 hours with fecal particles (*p* = 0.0179; S4 Table). We found that CVB3 incubated in artificial saliva without particles had a faster decay rate of −0.20 hr^-1^ compared to artificial saliva with particles (decay rate of −0.13 hr^-1^) (S1 Table).

We also incubated CVB3 in human saliva either with or without fecal particles at 37 °C then measure viruses at 0, 2, 5, 24, and 48 hours (Fig 3b). In both conditions, CVB3 titers declined over time with similar titers maintained during the first 2 hours (*p* = 0.8353; S5 Table). Beyond 2 hours, significant reductions in viral titers were observed relative to the initial 0-hour point (*p* < 0.0001). The decay rate was faster in saliva without fecal particles compared to saliva with fecal particles (Specific decay rates are presented in S1 Table). These results suggest that fecal particles offer stabilization of CVB3 in both artificial saliva and human saliva.

### Impact of fecal particles on AdV41 stability in human and artificial saliva

**Fig 4:**
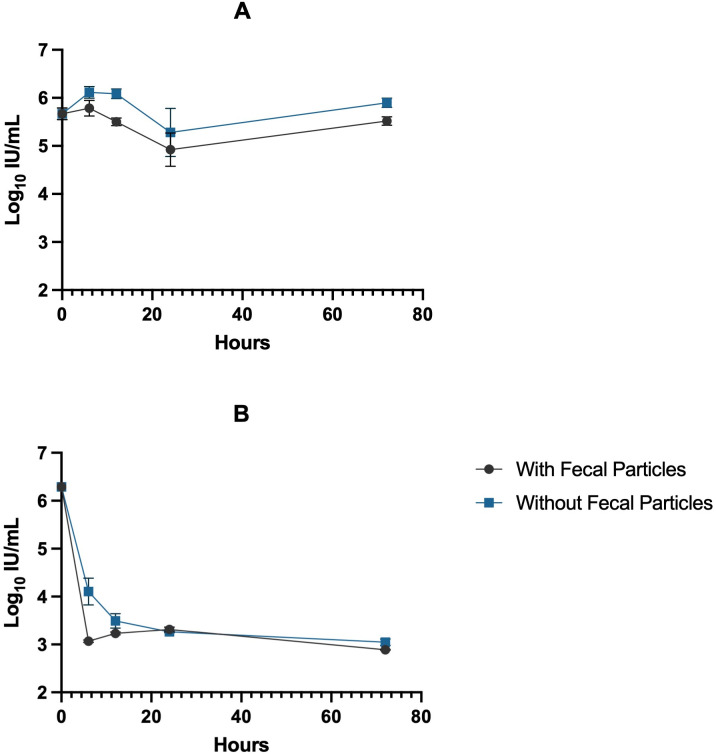
Decay of AdV41 in artificial saliva (A) and human saliva (B). AdV41 was incubated at 37 °C for 72 hours in the presence and absence of fecal particles represented as log₁₀IU/ mL (mean ± SD, n = 3). All time points were tested, however, values below the detection limit were not plotted on the graph.

To assess the impact of fecal particles on adenovirus stability, AdV41 was incubated in artificial saliva with or without fecal matter at 37°C (Fig 4a). At early time points (0–5 hours), there were no significant differences between the two conditions (*p* > 0.1; S6 Table). However, by 24 hours, AdV41 incubated with fecal particles exhibited slightly higher titers compared to the initial 0 h measurement without fecal particles (*p* = 0.0132; S6 Table) as well as relative to the corresponding 2 hours without fecal particles and 24 hours samples with and without fecal particles (*p* = 0.0064 and < 0.0001, respectively; S6 Table). The decay rate of AdV41 in artificial saliva with fecal particles and without fecal particles were the same (−0.01 hr^-1^) (S1 Table). Similarly, we assessed AdV41 in human saliva in the presence of fecal particles and without fecal particles over 72-hour period (Fig 4b). Both samples had equal viral titers at 0 hours (*p* > 0.9999; S7 Table). Notably, at 2 hours, there was significant viral titers lower in saliva containing fecal particles compared to saliva without particles (*p* < 0.0001; S7 Table), indicating accelerated inactivation in the presence of particles. However, by 5 hours onward, no significant differences were observed between the two matrices (*p* > 0.05; S7 Table), and by 72 hours, there was no significant difference in titers between AdV41 in human saliva with or without fecal particles (*p* = 0.9839; S7 Table). The decay rate was faster in human saliva (−0.06 hr^-1^) compared to artificial saliva (−0.05 hr^-1^) confirming that human saliva promotes more rapid inactivation of AdV41 regardless of the presence of fecal material (Specific decay rates are presented in S1 Table). As AdV41 exhibits extended environmental persistence, we evaluated its long-term stability following a 3-week incubation in human saliva with and without fecal particles (S1 Fig). While AdV41 remained stable in PBS, viral titers were significantly lower in human saliva containing fecal particles compared to saliva without particles (*p* < 0.001). These results suggest that, unlike CVB3, fecal particles may enhance viral inactivation rather than protection, potentially due to differential adsorption or aggregation effects that limit viral recovery.

## 4.0 Discussion

### Comparison with other studies done in saliva

Our study demonstrated that enteric viruses, CVB3 and AdV41, experience accelerated decay in human saliva, with CVB3 becoming undetectable in most replicates by 24−48 hours and AdV41 showing a more gradual decline over 72 hours. Although there is limited evidence on enteric virus decay in human saliva, existing literature provides evidence supporting the presence of enteric viruses in human saliva. For instance, Norovirus RNA has been detected in saliva samples collected during community outbreaks, indicating that enteric viruses may persist in saliva long enough to serve as potential biomarkers of recent infection [20]. Similarly, salivary detection of pathogen-specific antibodies and antigens has been employed to estimate exposure to enteric viruses, further reinforcing the utility of saliva as a non-invasive matrix for surveillance and exposure assessment [21,46]. In vivo, Ghosh et al. [47] demonstrated that murine norovirus 1 (MNV-1) and Rotavirus could be orally transmitted in mice, with viral shedding in saliva persisting for up to three weeks post-inoculation. While these studies have described viral presence or shedding in saliva, our study is among the first to characterize the decay kinetics of human enteric viruses in both artificial and human saliva matrices, providing insights into viral persistence and potential environmental risk of infection.

In contrast, prior studies have consistently examined both the detection and decay of enveloped respiratory viruses, such as Influenza A virus and SARS-CoV-2 in saliva, with findings showing that these viruses tend to decay more rapidly under ambient conditions than what we have observed for the tested enteric viruses [48]. Studies have demonstrated that Influenza A virus undergoes rapid decay in saliva droplets, particularly under intermediate relative humidity (IRH), whereas airway surface liquid droplets are more protective and retain infectivity over time [49]. Similarly, murine coronavirus and vesicular stomatitis virus (VSV) have shown fast decay in saliva under IRH conditions, suggesting that saliva presents a hostile extracellular environment for enveloped viruses in controlled humidity [50,51]. In contrast, previous work with bacteriophage Phi6 in saliva microdroplets suggests that control for relative humidity does not directly determine viral stability and viability [52].

While previous studies have investigated the effect of relative humidity on viral decay in saliva microdroplets, our experiments were conducted under constant laboratory incubation conditions. Therefore, our findings reflect the influence of biological and biochemical components within saliva on the stability of non-enveloped enteric viruses like CVB3 and AdV41.

Additionally, persistent oral viruses including herpes simplex virus 1 (HSV-1) and Epstein-Barr virus (EBV), often shed in saliva asymptomatically. These viruses evade antiviral immune responses and establish latency in host epithelial and immune cells supporting their extended persistence in saliva [53]. While relevant, such mechanisms differ from the decay processes observed in our enteric virus study, indicating that the persistence of viruses in saliva is highly virus-specific and influenced by both environmental and biological conditions. However, despite this persistence, studies have reported that HSV-1 infectivity decreases substantially within hours when exposed to Lactoferrin in saliva samples ex vivo [54].

### Virus stability in Artificial Saliva vs. Human Saliva

Our findings demonstrated that enteric viruses exhibited significantly greater stability in artificial saliva compared to human saliva. Viral titers declined more rapidly in human saliva in our study where by 24 and 48 hours were completely inactivated. Similarly findings from a study on SARS-CoV-2, reported a T90 (time for 1-log reduction) of only 1.8 hours in human saliva, compared to 9.1 hours in culture medium during UV inactivation although their study did not systematically control for or stratify by humidity levels [55], a difference likely attributable to the compositional complexity and biological variability of natural saliva [56]. Artificial saliva is a chemically defined formulation typically containing stabilizing agents such as mucins, glycerin, and carboxymethyl cellulose. These components are designed to replicate the viscosity of human saliva, and often include preservatives such as methyl- or propylparaben to maintain a neutral pH condition that may promote enhanced stability of viral capsids [57]. Notably, artificial saliva lacks active enzymes, immune mediators, and microbial constituents found in natural saliva, which are known to play critical roles in the degradation of viral particles [56].

In contrast, human saliva harbors a diverse microbiota and an array of innate immune components, including lysozyme, lactoferrin, proline-rich proteins, interferons, and immunoglobulins (IgA, IgG, and IgM), which can neutralize viruses by binding to capsid proteins or disrupting viral structure [7]. Furthermore, proteolytic enzymes present in human saliva can actively degrade viral capsids, thereby diminishing viral infectivity [56,58]. The biochemical composition of human saliva also varies depending on an individual’s nutritional status, hydration level, circadian rhythm, and age, factors that influence pH and ionic strength and may further destabilize viral particles [59]. These factors would likely contribute to enteric viruses being less stable in human saliva compared to artificial saliva.

### Protective role of fecal particles and oral bacteria

Fecal matter serves as a complex biological matrix rich in proteins, lipids, mucopolysaccharides, amino acids, organic acids, sugars, and a broad spectrum of microbial constituents [60,61]. These components can physically entrap viral particles, forming protective microenvironments that shield viruses from environmental stressors such as temperature fluctuations, desiccation, and enzymatic degradation [24]. Oral introduction of fecal-contaminated material is through the traditional fecal-oral transmission routes, commonly via inadequate hand hygiene or ingestion of contaminated water, food, or fomites [62]. It poses significant public health risks, by facilitating the transfer of enteric viruses into the oral cavity in a protective manner [63].

Organic and particulate-rich matrices such as feces have been shown to prolong viral viability through interactions between viruses and components such as lipids, proteins, and extracellular polymeric substances (EPS) forming virus-particle complexes that enhance capsid integrity and preserve infectivity [64]. These interactions reduce structural degradation, effectively stabilizing viral particles over extended periods even when exposed to major antimicrobial components of saliva [24]. Consistent with this, our findings showed that the presence of fecal particles significantly enhanced the persistence of CVB3 in both artificial and human saliva matrices.

Additionally, our study demonstrated that oral bacteria contribute to viral stability. Among the tested strains, *Streptococcus downei* showed the most notable stabilizing effect, while *Bacillus subtilis* (a gut and environmental bacteria known to enhance viral persistence) [65] and *Streptococcus mutans* showed moderate enhancement. This finding is among the first to suggest that oral bacteria may contribute to the persistence and infectivity of viruses in the human oral cavity. This is similar to intestinal bacteria which has been shown to facilitate the persistence of viruses for example, the infectivity of human astrovirus has been shown to be significantly enhanced in the presence of *Escherichia coli* [66]. Such effects are mediated by bacterial surface molecules like lipopolysaccharides (LPS) and peptidoglycans (PGN), which bind to viral capsids and protect them from conformational changes and premature genome release [28,67]. These interactions may stabilize viral structure, prevent RNA ejection, and shield the virion from degradation, ultimately enhancing viral survival in the oral environment [28,68].Though both fecal particles and oral bacteria can enhance viral stability, fecal particles are better able at protecting viruses when in the presence of human saliva (Fig 2a). This could possibly be due to the greater shielding or stabilizing ability of fecal particles. Fecal particles could also have greater stability than oral bacteria in saliva allowing them to protect enteric viruses longer. Additionally, since only the volume added of fecal particles or oral bacteria was standardized instead of particle number, it is difficult to determine the exact effect of each protective component. Future studies should explore parsing out the different aspects of human fecal material and determining the exact effect on viral stability each component of fecal material may have so a direct comparison between oral bacteria can be made. Nevertheless, the findings in this study offer novel insight into the protective role of these two distinct contributors, oral bacteria and fecal matter, in saliva.

### Differential Decay Rates of AdV41 and CVB3

Our study revealed a marked difference in the decay kinetics of AdV41 and CVB3 under similar incubation conditions. AdV41, a non-enveloped double-stranded DNA virus, demonstrated significantly slower decay rates than CVB3, a non-enveloped single-stranded RNA virus, particularly in artificial saliva. This finding aligns with prior literature indicating that DNA viruses tend to exhibit greater environmental stability than RNA viruses, largely due to the inherent chemical resilience and structural robustness of their genomes [69].

AdV41’s high stability is further attributed to its complex capsid architecture, which comprises at least nine structural proteins including hexon, penton base, and fiber proteins that confer protection against environmental stressors and enzymatic degradation [70]. These structural elements likely contribute to the virus’s resistance to inactivation, especially in chemically defined environments such as artificial saliva, which contains stabilizing agents and maintains a near-neutral pH [57]. Conversely, CVB3, although also non-enveloped, possesses a smaller and less complex capsid that encases a single-stranded RNA genome, which is more prone to hydrolysis and degradation [71]. Its structural simplicity makes it more susceptible to the actions of proteolytic enzymes and unfavorable physicochemical conditions, resulting in a more rapid decline in viral infectivity over time [71]. Several studies have also highlighted AdV41’s DNA repair mechanisms as a reason for its extended environmental survivability which could lead to its persistence in saliva [72,73].

### Enteric virus growth in saliva-associated cell lines

Recent studies have expanded our understanding of enteric virus-host interactions by demonstrating that salivary gland-derived cell lines can support the replication of enteric viruses traditionally associated with the gastrointestinal tract [47]. Building on these findings, Kosgei et al. [74] demonstrated that CVB3 and AdV41 can also replicate in NS-SV-AC (acinar) cells to increases over 2 logs. When comparing these log increases in saliva cells to the current study’s log decreases, decay in saliva would have a significantly lower reduction rate. These findings potentially show how certain viruses can survive the oral cavity and initiate infection in the lower parts of the gastrointestinal tract.

### Limitations

A major limitation of the current study is that it was an in vitro experiment, which may not fully replicate the complexity of the in vivo oral environment, including host immune responses and dynamic salivary flow. Additionally, the study was limited to only two different types of enteric viruses, CVB3 and AdV41, which limits generalizability of our findings across all enteric viruses. The study mainly controlled temperature but did not account for relative humidity similar to other studies [49]. Ambient environmental factors such as humidity and temperature has been shown in some studies to influence viral decay in saliva droplets and could significantly alter virus stability but as our study was done with larger volumes of saliva, results still provide significant insights. Future research should incorporate a wider range of viruses, utilize in vivo models, systematically evaluate how salivary composition, and environmental variables such as humidity and temperature affect viral persistence and potential for transmission.

## 5.0 Conclusion

This study provides significant findings into the factors influencing enteric virus persistence in human and artificial saliva. Fecal particles significantly enhance viral stability compared to oral bacteria, and artificial saliva generally supports longer viral survival than human saliva. These findings highlight the potential role of saliva as a medium for viral transmission and emphasize the need for continued research on enteric virus dynamics in saliva.

## Supporting information

S1 Fig Concentration of AdV41 after 21 days of incubation in Artificial saliva. (DOCX)

S1 Table Average calculated decay rates for each of the conditions tested for AdV41 and CVB3. (DOCX)

S2 Table Multiple comparison’s statistical test for all points in Fig 1A. (DOCX)

S3 Table Multiple comparison’s statistical test for all points in Fig 1B. (DOCX)

S4 Table Multiple comparison’s statistical test for all points in Fig 3A. (DOCX)

S5 Table Multiple comparison’s statistical test for all points in Fig 3B. (DOCX)

S6 Table Multiple comparison’s statistical test for all points in Fig 4A. (DOCX)

S7 Table Multiple comparison’s statistical test for all points in Fig 4B. (DOCX)

S8 Table Statistical comparisons for Fig 2A. (DOCX)

S9 Table Statistical comparisons for Fig 2C. (DOCX)

S10 Table Data supporting Figs 1-4. (DOCX)
