# Supplementary material for: Persistence of human enteric viruses in artificial and human saliva
Source: PLoS One. 2025 Dec 26;20(12):e0339724. doi: 10.1371/journal.pone.0339724 (PMC12742735; doi:10.1371/journal.pone.0339724)
Supplement: S1 Fig — (DOCX) [file pone.0339724.s001.docx]

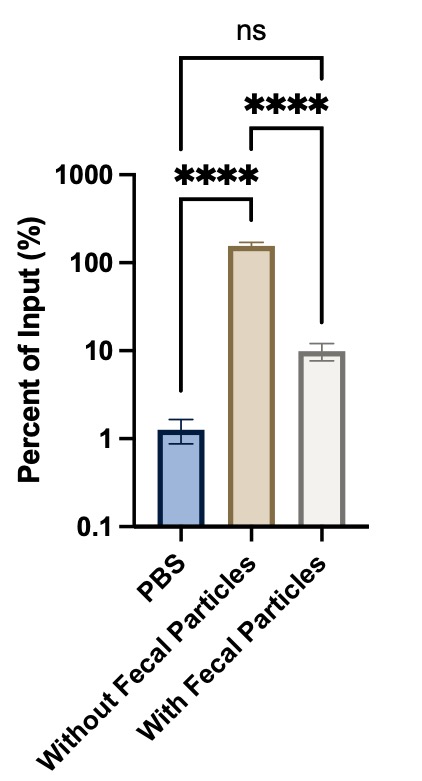


**Figure S1**: Concentration of AdV41 after 21 days of incubation in Artificial saliva. Data represented as percent of input (%) with statistical significance represented as ns (not significant), and *****p* < 0.0001.
