# Supplementary material for: Persistence of human enteric viruses in artificial and human saliva
Source: PLoS One. 2025 Dec 26;20(12):e0339724. doi: 10.1371/journal.pone.0339724 (PMC12742735; doi:10.1371/journal.pone.0339724)
Supplement: S1 Table — (DOCX) [file pone.0339724.s002.docx]

**Table S1**: Average calculated decay rates for each of the conditions tested for AdV41 and CVB3.

| Pathogen | Condition | Average k_obs_ (hr^-1^) | SD | R^2^ |
| --- | --- | --- | --- | --- |
| CVB3 | In PBS | -0.10 | 0.05 | 0.59 |
|  | In Artificial saliva without particles | -0.20 | 0.14 | 0.51 |
|  | In Artificial saliva with particles | -0.13 | 0.03 | 0.83 |
|  | In human saliva without particles | -1.13 | 0.41 | 0.88 |
|  | In human saliva with particles | -0.16 | 0.12 | 0.49 |
| AdV41 | In PBS | -0.03 | 0.02 | 0.51 |
|  | In Artificial saliva without particles | -0.01 | 0.02 | 0.14 |
|  | In Artificial saliva with particles | -0.01 | 0.02 | 0.15 |
|  | In human saliva without particles | -0.06 | 0.05 | 0.34 |
|  | In human saliva with particles | -0.05 | 0.06 | 0.20 |
