# Supplementary material for: Persistence of human enteric viruses in artificial and human saliva
Source: PLoS One. 2025 Dec 26;20(12):e0339724. doi: 10.1371/journal.pone.0339724 (PMC12742735; doi:10.1371/journal.pone.0339724)
Supplement: S2 Table — (DOCX) [file pone.0339724.s003.docx]

**Table S2:** Multiple comparison’s statistical test for all points in Figure 1A.

| **Tukey's multiple comparisons test** | **Predicted (LS) Mean diff.** | **95.00% CI of diff.** | **Below threshold?** | **Summary** | **Adjusted P Value** |
| --- | --- | --- | --- | --- | --- |
|  |  |  |  |  |  |
| 0:PBS vs. 0:Artificial Saliva | 0.2464 | -0.05315 to 0.5459 | No | ns | 0.2085 |
| 0:PBS vs. 0:Human Saliva | 0.7146 | 0.3368 to 1.092 | Yes | **** | <0.0001 |
| 0:PBS vs. 2:PBS | 0.7773 | 0.4485 to 1.106 | Yes | **** | <0.0001 |
| 0:PBS vs. 2:Artificial Saliva | 1.024 | 0.5809 to 1.466 | Yes | **** | <0.0001 |
| 0:PBS vs. 2:Human Saliva | 1.492 | 0.9370 to 2.047 | Yes | **** | <0.0001 |
| 0:PBS vs. 5:PBS | 2.300 | 1.894 to 2.707 | Yes | **** | <0.0001 |
| 0:PBS vs. 5:Artificial Saliva | 2.547 | 2.008 to 3.085 | Yes | **** | <0.0001 |
| 0:PBS vs. 5:Human Saliva | 3.015 | 2.417 to 3.612 | Yes | **** | <0.0001 |
| 0:PBS vs. 24:PBS | 2.446 | 2.028 to 2.863 | Yes | **** | <0.0001 |
| 0:PBS vs. 24:Artificial Saliva | 2.692 | 2.164 to 3.220 | Yes | **** | <0.0001 |
| 0:PBS vs. 48:PBS | 2.673 | 1.860 to 3.486 | Yes | **** | <0.0001 |
| 0:Artificial Saliva vs. 0:Human Saliva | 0.4682 | 0.08047 to 0.8559 | Yes | ** | 0.0063 |
| 0:Artificial Saliva vs. 2:PBS | 0.5309 | 0.08413 to 0.9777 | Yes | ** | 0.0077 |
| 0:Artificial Saliva vs. 2:Artificial Saliva | 0.7773 | 0.4485 to 1.106 | Yes | **** | <0.0001 |
| 0:Artificial Saliva vs. 2:Human Saliva | 1.245 | 0.6822 to 1.809 | Yes | **** | <0.0001 |
| 0:Artificial Saliva vs. 5:PBS | 2.054 | 1.585 to 2.523 | Yes | **** | <0.0001 |
| 0:Artificial Saliva vs. 5:Artificial Saliva | 2.300 | 1.894 to 2.707 | Yes | **** | <0.0001 |
| 0:Artificial Saliva vs. 5:Human Saliva | 2.768 | 2.194 to 3.343 | Yes | **** | <0.0001 |
| 0:Artificial Saliva vs. 24:PBS | 2.199 | 1.701 to 2.698 | Yes | **** | <0.0001 |
| 0:Artificial Saliva vs. 24:Artificial Saliva | 2.446 | 2.028 to 2.863 | Yes | **** | <0.0001 |
| 0:Artificial Saliva vs. 48:PBS | 2.427 | 1.612 to 3.241 | Yes | **** | <0.0001 |
| 0:Human Saliva vs. 2:PBS | 0.06275 | -0.3775 to 0.5030 | No | ns | >0.9999 |
| 0:Human Saliva vs. 2:Artificial Saliva | 0.3091 | -0.1377 to 0.7560 | No | ns | 0.4547 |
| 0:Human Saliva vs. 2:Human Saliva | 0.7773 | 0.4485 to 1.106 | Yes | **** | <0.0001 |
| 0:Human Saliva vs. 5:PBS | 1.586 | 1.077 to 2.094 | Yes | **** | <0.0001 |
| 0:Human Saliva vs. 5:Artificial Saliva | 1.832 | 1.283 to 2.381 | Yes | **** | <0.0001 |
| 0:Human Saliva vs. 5:Human Saliva | 2.300 | 1.894 to 2.707 | Yes | **** | <0.0001 |
| 0:Human Saliva vs. 24:PBS | 1.731 | 1.255 to 2.208 | Yes | **** | <0.0001 |
| 0:Human Saliva vs. 24:Artificial Saliva | 1.977 | 1.477 to 2.478 | Yes | **** | <0.0001 |
| 0:Human Saliva vs. 48:PBS | 1.958 | 1.133 to 2.784 | Yes | **** | <0.0001 |
| 2:PBS vs. 2:Artificial Saliva | 0.2464 | -0.05315 to 0.5459 | No | ns | 0.2085 |
| 2:PBS vs. 2:Human Saliva | 0.7146 | 0.3368 to 1.092 | Yes | **** | <0.0001 |
| 2:PBS vs. 5:PBS | 1.523 | 1.099 to 1.947 | Yes | **** | <0.0001 |
| 2:PBS vs. 5:Artificial Saliva | 1.769 | 1.216 to 2.323 | Yes | **** | <0.0001 |
| 2:PBS vs. 5:Human Saliva | 2.237 | 1.677 to 2.798 | Yes | **** | <0.0001 |
| 2:PBS vs. 24:PBS | 1.668 | 1.248 to 2.089 | Yes | **** | <0.0001 |
| 2:PBS vs. 24:Artificial Saliva | 1.915 | 1.382 to 2.447 | Yes | **** | <0.0001 |
| 2:PBS vs. 48:PBS | 1.896 | 1.081 to 2.711 | Yes | **** | <0.0001 |
| 2:Artificial Saliva vs. 2:Human Saliva | 0.4682 | 0.08047 to 0.8559 | Yes | ** | 0.0063 |
| 2:Artificial Saliva vs. 5:PBS | 1.277 | 0.7941 to 1.759 | Yes | **** | <0.0001 |
| 2:Artificial Saliva vs. 5:Artificial Saliva | 1.523 | 1.099 to 1.947 | Yes | **** | <0.0001 |
| 2:Artificial Saliva vs. 5:Human Saliva | 1.991 | 1.457 to 2.525 | Yes | **** | <0.0001 |
| 2:Artificial Saliva vs. 24:PBS | 1.422 | 0.9226 to 1.921 | Yes | **** | <0.0001 |
| 2:Artificial Saliva vs. 24:Artificial Saliva | 1.668 | 1.248 to 2.089 | Yes | **** | <0.0001 |
| 2:Artificial Saliva vs. 48:PBS | 1.649 | 0.8341 to 2.465 | Yes | **** | <0.0001 |
| 2:Human Saliva vs. 5:PBS | 0.8084 | 0.2336 to 1.383 | Yes | *** | 0.0007 |
| 2:Human Saliva vs. 5:Artificial Saliva | 1.055 | 0.4426 to 1.667 | Yes | **** | <0.0001 |
| 2:Human Saliva vs. 5:Human Saliva | 1.523 | 1.099 to 1.947 | Yes | **** | <0.0001 |
| 2:Human Saliva vs. 24:PBS | 0.9537 | 0.4182 to 1.489 | Yes | **** | <0.0001 |
| 2:Human Saliva vs. 24:Artificial Saliva | 1.200 | 0.6419 to 1.758 | Yes | **** | <0.0001 |
| 2:Human Saliva vs. 48:PBS | 1.181 | 0.3203 to 2.042 | Yes | *** | 0.0010 |
| 5:PBS vs. 5:Artificial Saliva | 0.2464 | -0.05315 to 0.5459 | No | ns | 0.2085 |
| 5:PBS vs. 5:Human Saliva | 0.7146 | 0.3368 to 1.092 | Yes | **** | <0.0001 |
| 5:PBS vs. 24:PBS | 0.1454 | -0.3435 to 0.6342 | No | ns | 0.9969 |
| 5:PBS vs. 24:Artificial Saliva | 0.3918 | -0.1643 to 0.9478 | No | ns | 0.4265 |
| 5:PBS vs. 48:PBS | 0.3728 | -0.4707 to 1.216 | No | ns | 0.9348 |
| 5:Artificial Saliva vs. 5:Human Saliva | 0.4682 | 0.08047 to 0.8559 | Yes | ** | 0.0063 |
| 5:Artificial Saliva vs. 24:PBS | -0.1010 | -0.6910 to 0.4890 | No | ns | >0.9999 |
| 5:Artificial Saliva vs. 24:Artificial Saliva | 0.1454 | -0.3435 to 0.6342 | No | ns | 0.9969 |
| 5:Artificial Saliva vs. 48:PBS | 0.1264 | -0.7389 to 0.9918 | No | ns | >0.9999 |
| 5:Human Saliva vs. 24:PBS | -0.5692 | -1.153 to 0.01476 | No | ns | 0.0627 |
| 5:Human Saliva vs. 24:Artificial Saliva | -0.3228 | -0.8965 to 0.2509 | No | ns | 0.7481 |
| 5:Human Saliva vs. 48:PBS | -0.3418 | -1.225 to 0.5418 | No | ns | 0.9742 |
| 24:PBS vs. 24:Artificial Saliva | 0.2464 | -0.05315 to 0.5459 | No | ns | 0.2085 |
| 24:PBS vs. 48:PBS | 0.2274 | -0.6171 to 1.072 | No | ns | 0.9987 |
| 24:Artificial Saliva vs. 48:PBS | -0.01895 | -0.8740 to 0.8361 | No | ns | >0.9999 |
