# Supplementary material for: Persistence of human enteric viruses in artificial and human saliva
Source: PLoS One. 2025 Dec 26;20(12):e0339724. doi: 10.1371/journal.pone.0339724 (PMC12742735; doi:10.1371/journal.pone.0339724)
Supplement: S3 Table — (DOCX) [file pone.0339724.s004.docx]

**Table S3:** Multiple comparison’s statistical test for all points in Figure 1B.

| **Tukey's multiple comparisons test** | **Mean diff.** | **95.00% CI of diff.** | **Below threshold?** | **Summary** | **Adjusted P Value** |
| --- | --- | --- | --- | --- | --- |
|  |  |  |  |  |  |
| 0:PBS vs. 0:Artificial Saliva | 0.000 | -0.7626 to 0.7626 | No | ns | >0.9999 |
| 0:PBS vs. 0:Human Saliva | -0.6200 | -1.383 to 0.1426 | No | ns | 0.2120 |
| 0:PBS vs. 2:PBS | 0.2000 | -0.5626 to 0.9626 | No | ns | 0.9995 |
| 0:PBS vs. 2:Artificial Saliva | -0.4433 | -1.206 to 0.3193 | No | ns | 0.6991 |
| 0:PBS vs. 2:Human Saliva | 1.563 | 0.8007 to 2.326 | Yes | **** | <0.0001 |
| 0:PBS vs. 5:PBS | 0.1600 | -0.6026 to 0.9226 | No | ns | >0.9999 |
| 0:PBS vs. 5:Artificial Saliva | -0.4167 | -1.179 to 0.3460 | No | ns | 0.7757 |
| 0:PBS vs. 5:Human Saliva | 2.177 | 1.414 to 2.939 | Yes | **** | <0.0001 |
| 0:PBS vs. 24:PBS | 0.5467 | -0.2160 to 1.309 | No | ns | 0.3838 |
| 0:PBS vs. 24:Artificial Saliva | 0.3900 | -0.3726 to 1.153 | No | ns | 0.8428 |
| 0:PBS vs. 24:Human Saliva | 2.407 | 1.644 to 3.169 | Yes | **** | <0.0001 |
| 0:PBS vs. 72:PBS | 0.7100 | -0.05262 to 1.473 | No | ns | 0.0884 |
| 0:PBS vs. 72:Artificial Saliva | -0.2267 | -0.9893 to 0.5360 | No | ns | 0.9981 |
| 0:PBS vs. 72:Human Saliva | 2.623 | 1.861 to 3.386 | Yes | **** | <0.0001 |
| 0:Artificial Saliva vs. 0:Human Saliva | -0.6200 | -1.383 to 0.1426 | No | ns | 0.2120 |
| 0:Artificial Saliva vs. 2:PBS | 0.2000 | -0.5626 to 0.9626 | No | ns | 0.9995 |
| 0:Artificial Saliva vs. 2:Artificial Saliva | -0.4433 | -1.206 to 0.3193 | No | ns | 0.6991 |
| 0:Artificial Saliva vs. 2:Human Saliva | 1.563 | 0.8007 to 2.326 | Yes | **** | <0.0001 |
| 0:Artificial Saliva vs. 5:PBS | 0.1600 | -0.6026 to 0.9226 | No | ns | >0.9999 |
| 0:Artificial Saliva vs. 5:Artificial Saliva | -0.4167 | -1.179 to 0.3460 | No | ns | 0.7757 |
| 0:Artificial Saliva vs. 5:Human Saliva | 2.177 | 1.414 to 2.939 | Yes | **** | <0.0001 |
| 0:Artificial Saliva vs. 24:PBS | 0.5467 | -0.2160 to 1.309 | No | ns | 0.3838 |
| 0:Artificial Saliva vs. 24:Artificial Saliva | 0.3900 | -0.3726 to 1.153 | No | ns | 0.8428 |
| 0:Artificial Saliva vs. 24:Human Saliva | 2.407 | 1.644 to 3.169 | Yes | **** | <0.0001 |
| 0:Artificial Saliva vs. 72:PBS | 0.7100 | -0.05262 to 1.473 | No | ns | 0.0884 |
| 0:Artificial Saliva vs. 72:Artificial Saliva | -0.2267 | -0.9893 to 0.5360 | No | ns | 0.9981 |
| 0:Artificial Saliva vs. 72:Human Saliva | 2.623 | 1.861 to 3.386 | Yes | **** | <0.0001 |
| 0:Human Saliva vs. 2:PBS | 0.8200 | 0.05738 to 1.583 | Yes | * | 0.0259 |
| 0:Human Saliva vs. 2:Artificial Saliva | 0.1767 | -0.5860 to 0.9393 | No | ns | 0.9999 |
| 0:Human Saliva vs. 2:Human Saliva | 2.183 | 1.421 to 2.946 | Yes | **** | <0.0001 |
| 0:Human Saliva vs. 5:PBS | 0.7800 | 0.01738 to 1.543 | Yes | * | 0.0411 |
| 0:Human Saliva vs. 5:Artificial Saliva | 0.2033 | -0.5593 to 0.9660 | No | ns | 0.9994 |
| 0:Human Saliva vs. 5:Human Saliva | 2.797 | 2.034 to 3.559 | Yes | **** | <0.0001 |
| 0:Human Saliva vs. 24:PBS | 1.167 | 0.4040 to 1.929 | Yes | *** | 0.0003 |
| 0:Human Saliva vs. 24:Artificial Saliva | 1.010 | 0.2474 to 1.773 | Yes | ** | 0.0025 |
| 0:Human Saliva vs. 24:Human Saliva | 3.027 | 2.264 to 3.789 | Yes | **** | <0.0001 |
| 0:Human Saliva vs. 72:PBS | 1.330 | 0.5674 to 2.093 | Yes | **** | <0.0001 |
| 0:Human Saliva vs. 72:Artificial Saliva | 0.3933 | -0.3693 to 1.156 | No | ns | 0.8350 |
| 0:Human Saliva vs. 72:Human Saliva | 3.243 | 2.481 to 4.006 | Yes | **** | <0.0001 |
| 2:PBS vs. 2:Artificial Saliva | -0.6433 | -1.406 to 0.1193 | No | ns | 0.1713 |
| 2:PBS vs. 2:Human Saliva | 1.363 | 0.6007 to 2.126 | Yes | **** | <0.0001 |
| 2:PBS vs. 5:PBS | -0.04000 | -0.8026 to 0.7226 | No | ns | >0.9999 |
| 2:PBS vs. 5:Artificial Saliva | -0.6167 | -1.379 to 0.1460 | No | ns | 0.2184 |
| 2:PBS vs. 5:Human Saliva | 1.977 | 1.214 to 2.739 | Yes | **** | <0.0001 |
| 2:PBS vs. 24:PBS | 0.3467 | -0.4160 to 1.109 | No | ns | 0.9249 |
| 2:PBS vs. 24:Artificial Saliva | 0.1900 | -0.5726 to 0.9526 | No | ns | 0.9997 |
| 2:PBS vs. 24:Human Saliva | 2.207 | 1.444 to 2.969 | Yes | **** | <0.0001 |
| 2:PBS vs. 72:PBS | 0.5100 | -0.2526 to 1.273 | No | ns | 0.4915 |
| 2:PBS vs. 72:Artificial Saliva | -0.4267 | -1.189 to 0.3360 | No | ns | 0.7479 |
| 2:PBS vs. 72:Human Saliva | 2.423 | 1.661 to 3.186 | Yes | **** | <0.0001 |
| 2:Artificial Saliva vs. 2:Human Saliva | 2.007 | 1.244 to 2.769 | Yes | **** | <0.0001 |
| 2:Artificial Saliva vs. 5:PBS | 0.6033 | -0.1593 to 1.366 | No | ns | 0.2452 |
| 2:Artificial Saliva vs. 5:Artificial Saliva | 0.02667 | -0.7360 to 0.7893 | No | ns | >0.9999 |
| 2:Artificial Saliva vs. 5:Human Saliva | 2.620 | 1.857 to 3.383 | Yes | **** | <0.0001 |
| 2:Artificial Saliva vs. 24:PBS | 0.9900 | 0.2274 to 1.753 | Yes | ** | 0.0032 |
| 2:Artificial Saliva vs. 24:Artificial Saliva | 0.8333 | 0.07071 to 1.596 | Yes | * | 0.0221 |
| 2:Artificial Saliva vs. 24:Human Saliva | 2.850 | 2.087 to 3.613 | Yes | **** | <0.0001 |
| 2:Artificial Saliva vs. 72:PBS | 1.153 | 0.3907 to 1.916 | Yes | *** | 0.0004 |
| 2:Artificial Saliva vs. 72:Artificial Saliva | 0.2167 | -0.5460 to 0.9793 | No | ns | 0.9988 |
| 2:Artificial Saliva vs. 72:Human Saliva | 3.067 | 2.304 to 3.829 | Yes | **** | <0.0001 |
| 2:Human Saliva vs. 5:PBS | -1.403 | -2.166 to -0.6407 | Yes | **** | <0.0001 |
| 2:Human Saliva vs. 5:Artificial Saliva | -1.980 | -2.743 to -1.217 | Yes | **** | <0.0001 |
| 2:Human Saliva vs. 5:Human Saliva | 0.6133 | -0.1493 to 1.376 | No | ns | 0.2249 |
| 2:Human Saliva vs. 24:PBS | -1.017 | -1.779 to -0.2540 | Yes | ** | 0.0023 |
| 2:Human Saliva vs. 24:Artificial Saliva | -1.173 | -1.936 to -0.4107 | Yes | *** | 0.0003 |
| 2:Human Saliva vs. 24:Human Saliva | 0.8433 | 0.08071 to 1.606 | Yes | * | 0.0196 |
| 2:Human Saliva vs. 72:PBS | -0.8533 | -1.616 to -0.09071 | Yes | * | 0.0174 |
| 2:Human Saliva vs. 72:Artificial Saliva | -1.790 | -2.553 to -1.027 | Yes | **** | <0.0001 |
| 2:Human Saliva vs. 72:Human Saliva | 1.060 | 0.2974 to 1.823 | Yes | ** | 0.0013 |
| 5:PBS vs. 5:Artificial Saliva | -0.5767 | -1.339 to 0.1860 | No | ns | 0.3056 |
| 5:PBS vs. 5:Human Saliva | 2.017 | 1.254 to 2.779 | Yes | **** | <0.0001 |
| 5:PBS vs. 24:PBS | 0.3867 | -0.3760 to 1.149 | No | ns | 0.8504 |
| 5:PBS vs. 24:Artificial Saliva | 0.2300 | -0.5326 to 0.9926 | No | ns | 0.9978 |
| 5:PBS vs. 24:Human Saliva | 2.247 | 1.484 to 3.009 | Yes | **** | <0.0001 |
| 5:PBS vs. 72:PBS | 0.5500 | -0.2126 to 1.313 | No | ns | 0.3747 |
| 5:PBS vs. 72:Artificial Saliva | -0.3867 | -1.149 to 0.3760 | No | ns | 0.8504 |
| 5:PBS vs. 72:Human Saliva | 2.463 | 1.701 to 3.226 | Yes | **** | <0.0001 |
| 5:Artificial Saliva vs. 5:Human Saliva | 2.593 | 1.831 to 3.356 | Yes | **** | <0.0001 |
| 5:Artificial Saliva vs. 24:PBS | 0.9633 | 0.2007 to 1.726 | Yes | ** | 0.0045 |
| 5:Artificial Saliva vs. 24:Artificial Saliva | 0.8067 | 0.04404 to 1.569 | Yes | * | 0.0303 |
| 5:Artificial Saliva vs. 24:Human Saliva | 2.823 | 2.061 to 3.586 | Yes | **** | <0.0001 |
| 5:Artificial Saliva vs. 72:PBS | 1.127 | 0.3640 to 1.889 | Yes | *** | 0.0005 |
| 5:Artificial Saliva vs. 72:Artificial Saliva | 0.1900 | -0.5726 to 0.9526 | No | ns | 0.9997 |
| 5:Artificial Saliva vs. 72:Human Saliva | 3.040 | 2.277 to 3.803 | Yes | **** | <0.0001 |
| 5:Human Saliva vs. 24:PBS | -1.630 | -2.393 to -0.8674 | Yes | **** | <0.0001 |
| 5:Human Saliva vs. 24:Artificial Saliva | -1.787 | -2.549 to -1.024 | Yes | **** | <0.0001 |
| 5:Human Saliva vs. 24:Human Saliva | 0.2300 | -0.5326 to 0.9926 | No | ns | 0.9978 |
| 5:Human Saliva vs. 72:PBS | -1.467 | -2.229 to -0.7040 | Yes | **** | <0.0001 |
| 5:Human Saliva vs. 72:Artificial Saliva | -2.403 | -3.166 to -1.641 | Yes | **** | <0.0001 |
| 5:Human Saliva vs. 72:Human Saliva | 0.4467 | -0.3160 to 1.209 | No | ns | 0.6891 |
| 24:PBS vs. 24:Artificial Saliva | -0.1567 | -0.9193 to 0.6060 | No | ns | >0.9999 |
| 24:PBS vs. 24:Human Saliva | 1.860 | 1.097 to 2.623 | Yes | **** | <0.0001 |
| 24:PBS vs. 72:PBS | 0.1633 | -0.5993 to 0.9260 | No | ns | >0.9999 |
| 24:PBS vs. 72:Artificial Saliva | -0.7733 | -1.536 to -0.01071 | Yes | * | 0.0443 |
| 24:PBS vs. 72:Human Saliva | 2.077 | 1.314 to 2.839 | Yes | **** | <0.0001 |
| 24:Artificial Saliva vs. 24:Human Saliva | 2.017 | 1.254 to 2.779 | Yes | **** | <0.0001 |
| 24:Artificial Saliva vs. 72:PBS | 0.3200 | -0.4426 to 1.083 | No | ns | 0.9578 |
| 24:Artificial Saliva vs. 72:Artificial Saliva | -0.6167 | -1.379 to 0.1460 | No | ns | 0.2184 |
| 24:Artificial Saliva vs. 72:Human Saliva | 2.233 | 1.471 to 2.996 | Yes | **** | <0.0001 |
| 24:Human Saliva vs. 72:PBS | -1.697 | -2.459 to -0.9340 | Yes | **** | <0.0001 |
| 24:Human Saliva vs. 72:Artificial Saliva | -2.633 | -3.396 to -1.871 | Yes | **** | <0.0001 |
| 24:Human Saliva vs. 72:Human Saliva | 0.2167 | -0.5460 to 0.9793 | No | ns | 0.9988 |
| 72:PBS vs. 72:Artificial Saliva | -0.9367 | -1.699 to -0.1740 | Yes | ** | 0.0062 |
| 72:PBS vs. 72:Human Saliva | 1.913 | 1.151 to 2.676 | Yes | **** | <0.0001 |
| 72:Artificial Saliva vs. 72:Human Saliva | 2.850 | 2.087 to 3.613 | Yes | **** | <0.0001 |
