# Supplementary material for: Persistence of human enteric viruses in artificial and human saliva
Source: PLoS One. 2025 Dec 26;20(12):e0339724. doi: 10.1371/journal.pone.0339724 (PMC12742735; doi:10.1371/journal.pone.0339724)
Supplement: S4 Table — (DOCX) [file pone.0339724.s005.docx]

**Table S4:** Multiple comparison’s statistical test for all points in Figure 3A.

| **Tukey's multiple comparisons test** | **Predicted (LS) Mean diff.** | **95.00% CI of diff.** | **Below threshold?** | **Summary** | **Adjusted P Value** |
| --- | --- | --- | --- | --- | --- |
|  |  |  |  |  |  |
| 0:With Fecal Particles vs. 0:Without Fecal Particles | 0.2307 | -0.06954 to 0.5309 | No | ns | 0.2613 |
| 0:With Fecal Particles vs. 2:With Fecal Particles | 0.6383 | 0.2813 to 0.9952 | Yes | **** | <0.0001 |
| 0:With Fecal Particles vs. 2:Without Fecal Particles | 0.8689 | 0.4025 to 1.335 | Yes | **** | <0.0001 |
| 0:With Fecal Particles vs. 5:With Fecal Particles | 1.599 | 1.138 to 2.060 | Yes | **** | <0.0001 |
| 0:With Fecal Particles vs. 5:Without Fecal Particles | 1.830 | 1.240 to 2.420 | Yes | **** | <0.0001 |
| 0:With Fecal Particles vs. 24:With Fecal Particles | 2.580 | 2.142 to 3.017 | Yes | **** | <0.0001 |
| 0:With Fecal Particles vs. 24:Without Fecal Particles | 2.810 | 2.270 to 3.350 | Yes | **** | <0.0001 |
| 0:With Fecal Particles vs. 48:With Fecal Particles | 3.008 | 2.324 to 3.693 | Yes | **** | <0.0001 |
| 0:Without Fecal Particles vs. 2:With Fecal Particles | 0.4076 | -0.05878 to 0.8740 | No | ns | 0.1315 |
| 0:Without Fecal Particles vs. 2:Without Fecal Particles | 0.6383 | 0.2813 to 0.9952 | Yes | **** | <0.0001 |
| 0:Without Fecal Particles vs. 5:With Fecal Particles | 1.368 | 0.8608 to 1.876 | Yes | **** | <0.0001 |
| 0:Without Fecal Particles vs. 5:Without Fecal Particles | 1.599 | 1.138 to 2.060 | Yes | **** | <0.0001 |
| 0:Without Fecal Particles vs. 24:With Fecal Particles | 2.349 | 1.828 to 2.870 | Yes | **** | <0.0001 |
| 0:Without Fecal Particles vs. 24:Without Fecal Particles | 2.580 | 2.142 to 3.017 | Yes | **** | <0.0001 |
| 0:Without Fecal Particles vs. 48:With Fecal Particles | 2.778 | 2.093 to 3.462 | Yes | **** | <0.0001 |
| 2:With Fecal Particles vs. 2:Without Fecal Particles | 0.2307 | -0.06954 to 0.5309 | No | ns | 0.2613 |
| 2:With Fecal Particles vs. 5:With Fecal Particles | 0.9608 | 0.4997 to 1.422 | Yes | **** | <0.0001 |
| 2:With Fecal Particles vs. 5:Without Fecal Particles | 1.191 | 0.6017 to 1.781 | Yes | **** | <0.0001 |
| 2:With Fecal Particles vs. 24:With Fecal Particles | 1.941 | 1.504 to 2.379 | Yes | **** | <0.0001 |
| 2:With Fecal Particles vs. 24:Without Fecal Particles | 2.172 | 1.632 to 2.712 | Yes | **** | <0.0001 |
| 2:With Fecal Particles vs. 48:With Fecal Particles | 2.370 | 1.686 to 3.054 | Yes | **** | <0.0001 |
| 2:Without Fecal Particles vs. 5:With Fecal Particles | 0.7302 | 0.2225 to 1.238 | Yes | *** | 0.0007 |
| 2:Without Fecal Particles vs. 5:Without Fecal Particles | 0.9608 | 0.4997 to 1.422 | Yes | **** | <0.0001 |
| 2:Without Fecal Particles vs. 24:With Fecal Particles | 1.711 | 1.190 to 2.232 | Yes | **** | <0.0001 |
| 2:Without Fecal Particles vs. 24:Without Fecal Particles | 1.941 | 1.504 to 2.379 | Yes | **** | <0.0001 |
| 2:Without Fecal Particles vs. 48:With Fecal Particles | 2.139 | 1.455 to 2.824 | Yes | **** | <0.0001 |
| 5:With Fecal Particles vs. 5:Without Fecal Particles | 0.2307 | -0.06954 to 0.5309 | No | ns | 0.2613 |
| 5:With Fecal Particles vs. 24:With Fecal Particles | 0.9804 | 0.4569 to 1.504 | Yes | **** | <0.0001 |
| 5:With Fecal Particles vs. 24:Without Fecal Particles | 1.211 | 0.6373 to 1.785 | Yes | **** | <0.0001 |
| 5:With Fecal Particles vs. 48:With Fecal Particles | 1.409 | 0.6805 to 2.138 | Yes | **** | <0.0001 |
| 5:Without Fecal Particles vs. 24:With Fecal Particles | 0.7498 | 0.1179 to 1.382 | Yes | ** | 0.0095 |
| 5:Without Fecal Particles vs. 24:Without Fecal Particles | 0.9804 | 0.4569 to 1.504 | Yes | **** | <0.0001 |
| 5:Without Fecal Particles vs. 48:With Fecal Particles | 1.179 | 0.4195 to 1.938 | Yes | *** | 0.0002 |
| 24:With Fecal Particles vs. 24:Without Fecal Particles | 0.2307 | -0.06954 to 0.5309 | No | ns | 0.2613 |
| 24:With Fecal Particles vs. 48:With Fecal Particles | 0.4288 | -0.2974 to 1.155 | No | ns | 0.6077 |
| 24:Without Fecal Particles vs. 48:With Fecal Particles | 0.1981 | -0.5349 to 0.9312 | No | ns | 0.9933 |
