# Supplementary material for: Persistence of human enteric viruses in artificial and human saliva
Source: PLoS One. 2025 Dec 26;20(12):e0339724. doi: 10.1371/journal.pone.0339724 (PMC12742735; doi:10.1371/journal.pone.0339724)
Supplement: S5 Table — (DOCX) [file pone.0339724.s006.docx]

**Table S5:** Multiple comparison’s statistical test for all points in Figure 3B.

| **Tukey's multiple comparisons test** | **Predicted (LS) Mean diff.** | **95.00% CI of diff.** | **Below threshold?** | **Summary** | **Adjusted P Value** |
| --- | --- | --- | --- | --- | --- |
|  |  |  |  |  |  |
| 0:With Fecal Particles vs. 0:Without Fecal Particles | 0.1085 | -0.1523 to 0.3693 | No | ns | 0.8353 |
| 0:With Fecal Particles vs. 2:With Fecal Particles | 1.798 | 1.445 to 2.151 | Yes | **** | <0.0001 |
| 0:With Fecal Particles vs. 2:Without Fecal Particles | 1.907 | 1.452 to 2.361 | Yes | **** | <0.0001 |
| 0:With Fecal Particles vs. 5:With Fecal Particles | 2.176 | 1.861 to 2.492 | Yes | **** | <0.0001 |
| 0:With Fecal Particles vs. 5:Without Fecal Particles | 2.285 | 1.842 to 2.728 | Yes | **** | <0.0001 |
| 0:With Fecal Particles vs. 24:With Fecal Particles | 2.594 | 2.059 to 3.129 | Yes | **** | <0.0001 |
| 0:Without Fecal Particles vs. 2:With Fecal Particles | 1.690 | 1.266 to 2.113 | Yes | **** | <0.0001 |
| 0:Without Fecal Particles vs. 2:Without Fecal Particles | 1.798 | 1.445 to 2.151 | Yes | **** | <0.0001 |
| 0:Without Fecal Particles vs. 5:With Fecal Particles | 2.068 | 1.696 to 2.440 | Yes | **** | <0.0001 |
| 0:Without Fecal Particles vs. 5:Without Fecal Particles | 2.176 | 1.861 to 2.492 | Yes | **** | <0.0001 |
| 0:Without Fecal Particles vs. 24:With Fecal Particles | 2.486 | 1.950 to 3.021 | Yes | **** | <0.0001 |
| 2:With Fecal Particles vs. 2:Without Fecal Particles | 0.1085 | -0.1523 to 0.3693 | No | ns | 0.8353 |
| 2:With Fecal Particles vs. 5:With Fecal Particles | 0.3783 | -0.03057 to 0.7871 | No | ns | 0.0836 |
| 2:With Fecal Particles vs. 5:Without Fecal Particles | 0.4868 | -0.01397 to 0.9875 | No | ns | 0.0608 |
| 2:With Fecal Particles vs. 24:With Fecal Particles | 0.7960 | 0.2041 to 1.388 | Yes | ** | 0.0035 |
| 2:Without Fecal Particles vs. 5:With Fecal Particles | 0.2698 | -0.1989 to 0.7385 | No | ns | 0.5399 |
| 2:Without Fecal Particles vs. 5:Without Fecal Particles | 0.3783 | -0.03057 to 0.7871 | No | ns | 0.0836 |
| 2:Without Fecal Particles vs. 24:With Fecal Particles | 0.6875 | 0.08418 to 1.291 | Yes | * | 0.0179 |
| 5:With Fecal Particles vs. 5:Without Fecal Particles | 0.1085 | -0.1523 to 0.3693 | No | ns | 0.8353 |
| 5:With Fecal Particles vs. 24:With Fecal Particles | 0.4177 | -0.1456 to 0.9810 | No | ns | 0.2544 |
| 5:Without Fecal Particles vs. 24:With Fecal Particles | 0.3092 | -0.2794 to 0.8978 | No | ns | 0.6400 |
