# Supplementary material for: Persistence of human enteric viruses in artificial and human saliva
Source: PLoS One. 2025 Dec 26;20(12):e0339724. doi: 10.1371/journal.pone.0339724 (PMC12742735; doi:10.1371/journal.pone.0339724)
Supplement: S6 Table — (DOCX) [file pone.0339724.s007.docx]

**Table S6:** Multiple comparison’s statistical test for all points in Figure 4A.

| **Tukey's multiple comparisons test** | **Mean diff.** | **95.00% CI of diff.** | **Below threshold?** | **Summary** | **Adjusted P Value** |
| --- | --- | --- | --- | --- | --- |
|  |  |  |  |  |  |
| 0:With Fecal Particles vs. 0:Without Fecal Particles | 0.000 | -0.6332 to 0.6332 | No | ns | >0.9999 |
| 0:With Fecal Particles vs. 2:With Fecal Particles | -0.1167 | -0.7499 to 0.5165 | No | ns | 0.9995 |
| 0:With Fecal Particles vs. 2:Without Fecal Particles | -0.4433 | -1.077 to 0.1899 | No | ns | 0.3349 |
| 0:With Fecal Particles vs. 5:With Fecal Particles | 0.1667 | -0.4665 to 0.7999 | No | ns | 0.9931 |
| 0:With Fecal Particles vs. 5:Without Fecal Particles | -0.4167 | -1.050 to 0.2165 | No | ns | 0.4127 |
| 0:With Fecal Particles vs. 24:With Fecal Particles | 0.7467 | 0.1135 to 1.380 | Yes | * | 0.0132 |
| 0:With Fecal Particles vs. 24:Without Fecal Particles | 0.3900 | -0.2432 to 1.023 | No | ns | 0.4984 |
| 0:With Fecal Particles vs. 72:With Fecal Particles | 0.1533 | -0.4799 to 0.7865 | No | ns | 0.9962 |
| 0:With Fecal Particles vs. 72:Without Fecal Particles | -0.2267 | -0.8599 to 0.4065 | No | ns | 0.9501 |
| 0:Without Fecal Particles vs. 2:With Fecal Particles | -0.1167 | -0.7499 to 0.5165 | No | ns | 0.9995 |
| 0:Without Fecal Particles vs. 2:Without Fecal Particles | -0.4433 | -1.077 to 0.1899 | No | ns | 0.3349 |
| 0:Without Fecal Particles vs. 5:With Fecal Particles | 0.1667 | -0.4665 to 0.7999 | No | ns | 0.9931 |
| 0:Without Fecal Particles vs. 5:Without Fecal Particles | -0.4167 | -1.050 to 0.2165 | No | ns | 0.4127 |
| 0:Without Fecal Particles vs. 24:With Fecal Particles | 0.7467 | 0.1135 to 1.380 | Yes | * | 0.0132 |
| 0:Without Fecal Particles vs. 24:Without Fecal Particles | 0.3900 | -0.2432 to 1.023 | No | ns | 0.4984 |
| 0:Without Fecal Particles vs. 72:With Fecal Particles | 0.1533 | -0.4799 to 0.7865 | No | ns | 0.9962 |
| 0:Without Fecal Particles vs. 72:Without Fecal Particles | -0.2267 | -0.8599 to 0.4065 | No | ns | 0.9501 |
| 2:With Fecal Particles vs. 2:Without Fecal Particles | -0.3267 | -0.9599 to 0.3065 | No | ns | 0.7126 |
| 2:With Fecal Particles vs. 5:With Fecal Particles | 0.2833 | -0.3499 to 0.9165 | No | ns | 0.8406 |
| 2:With Fecal Particles vs. 5:Without Fecal Particles | -0.3000 | -0.9332 to 0.3332 | No | ns | 0.7950 |
| 2:With Fecal Particles vs. 24:With Fecal Particles | 0.8633 | 0.2301 to 1.497 | Yes | ** | 0.0032 |
| 2:With Fecal Particles vs. 24:Without Fecal Particles | 0.5067 | -0.1265 to 1.140 | No | ns | 0.1904 |
| 2:With Fecal Particles vs. 72:With Fecal Particles | 0.2700 | -0.3632 to 0.9032 | No | ns | 0.8730 |
| 2:With Fecal Particles vs. 72:Without Fecal Particles | -0.1100 | -0.7432 to 0.5232 | No | ns | 0.9997 |
| 2:Without Fecal Particles vs. 5:With Fecal Particles | 0.6100 | -0.02321 to 1.243 | No | ns | 0.0649 |
| 2:Without Fecal Particles vs. 5:Without Fecal Particles | 0.02667 | -0.6065 to 0.6599 | No | ns | >0.9999 |
| 2:Without Fecal Particles vs. 24:With Fecal Particles | 1.190 | 0.5568 to 1.823 | Yes | **** | <0.0001 |
| 2:Without Fecal Particles vs. 24:Without Fecal Particles | 0.8333 | 0.2001 to 1.467 | Yes | ** | 0.0046 |
| 2:Without Fecal Particles vs. 72:With Fecal Particles | 0.5967 | -0.03654 to 1.230 | No | ns | 0.0752 |
| 2:Without Fecal Particles vs. 72:Without Fecal Particles | 0.2167 | -0.4165 to 0.8499 | No | ns | 0.9617 |
| 5:With Fecal Particles vs. 5:Without Fecal Particles | -0.5833 | -1.217 to 0.04988 | No | ns | 0.0869 |
| 5:With Fecal Particles vs. 24:With Fecal Particles | 0.5800 | -0.05321 to 1.213 | No | ns | 0.0901 |
| 5:With Fecal Particles vs. 24:Without Fecal Particles | 0.2233 | -0.4099 to 0.8565 | No | ns | 0.9542 |
| 5:With Fecal Particles vs. 72:With Fecal Particles | -0.01333 | -0.6465 to 0.6199 | No | ns | >0.9999 |
| 5:With Fecal Particles vs. 72:Without Fecal Particles | -0.3933 | -1.027 to 0.2399 | No | ns | 0.4873 |
| 5:Without Fecal Particles vs. 24:With Fecal Particles | 1.163 | 0.5301 to 1.797 | Yes | **** | <0.0001 |
| 5:Without Fecal Particles vs. 24:Without Fecal Particles | 0.8067 | 0.1735 to 1.440 | Yes | ** | 0.0064 |
| 5:Without Fecal Particles vs. 72:With Fecal Particles | 0.5700 | -0.06321 to 1.203 | No | ns | 0.1002 |
| 5:Without Fecal Particles vs. 72:Without Fecal Particles | 0.1900 | -0.4432 to 0.8232 | No | ns | 0.9833 |
| 24:With Fecal Particles vs. 24:Without Fecal Particles | -0.3567 | -0.9899 to 0.2765 | No | ns | 0.6117 |
| 24:With Fecal Particles vs. 72:With Fecal Particles | -0.5933 | -1.227 to 0.03988 | No | ns | 0.0779 |
| 24:With Fecal Particles vs. 72:Without Fecal Particles | -0.9733 | -1.607 to -0.3401 | Yes | *** | 0.0008 |
| 24:Without Fecal Particles vs. 72:With Fecal Particles | -0.2367 | -0.8699 to 0.3965 | No | ns | 0.9362 |
| 24:Without Fecal Particles vs. 72:Without Fecal Particles | -0.6167 | -1.250 to 0.01654 | No | ns | 0.0602 |
| 72:With Fecal Particles vs. 72:Without Fecal Particles | -0.3800 | -1.013 to 0.2532 | No | ns | 0.5320 |
