# Supplementary material for: Persistence of human enteric viruses in artificial and human saliva
Source: PLoS One. 2025 Dec 26;20(12):e0339724. doi: 10.1371/journal.pone.0339724 (PMC12742735; doi:10.1371/journal.pone.0339724)
Supplement: S7 Table — (DOCX) [file pone.0339724.s008.docx]

**Table S7:** Multiple comparison’s statistical test for all points in Figure 4B.

| **Tukey's multiple comparisons test** | **Mean diff.** | **95.00% CI of diff.** | **Below threshold?** | **Summary** | **Adjusted P Value** |
| --- | --- | --- | --- | --- | --- |
|  |  |  |  |  |  |
| 0:With Fecal Particles vs. 0:Without Fecal Particles | 0.000 | -0.5251 to 0.5251 | No | ns | >0.9999 |
| 0:With Fecal Particles vs. 2:With Fecal Particles | 3.220 | 2.695 to 3.745 | Yes | **** | <0.0001 |
| 0:With Fecal Particles vs. 2:Without Fecal Particles | 2.183 | 1.658 to 2.708 | Yes | **** | <0.0001 |
| 0:With Fecal Particles vs. 5:With Fecal Particles | 3.057 | 2.532 to 3.582 | Yes | **** | <0.0001 |
| 0:With Fecal Particles vs. 5:Without Fecal Particles | 2.797 | 2.272 to 3.322 | Yes | **** | <0.0001 |
| 0:With Fecal Particles vs. 24:With Fecal Particles | 2.977 | 2.452 to 3.502 | Yes | **** | <0.0001 |
| 0:With Fecal Particles vs. 24:Without Fecal Particles | 3.027 | 2.502 to 3.552 | Yes | **** | <0.0001 |
| 0:With Fecal Particles vs. 72:With Fecal Particles | 3.400 | 2.875 to 3.925 | Yes | **** | <0.0001 |
| 0:With Fecal Particles vs. 72:Without Fecal Particles | 3.243 | 2.718 to 3.768 | Yes | **** | <0.0001 |
| 0:Without Fecal Particles vs. 2:With Fecal Particles | 3.220 | 2.695 to 3.745 | Yes | **** | <0.0001 |
| 0:Without Fecal Particles vs. 2:Without Fecal Particles | 2.183 | 1.658 to 2.708 | Yes | **** | <0.0001 |
| 0:Without Fecal Particles vs. 5:With Fecal Particles | 3.057 | 2.532 to 3.582 | Yes | **** | <0.0001 |
| 0:Without Fecal Particles vs. 5:Without Fecal Particles | 2.797 | 2.272 to 3.322 | Yes | **** | <0.0001 |
| 0:Without Fecal Particles vs. 24:With Fecal Particles | 2.977 | 2.452 to 3.502 | Yes | **** | <0.0001 |
| 0:Without Fecal Particles vs. 24:Without Fecal Particles | 3.027 | 2.502 to 3.552 | Yes | **** | <0.0001 |
| 0:Without Fecal Particles vs. 72:With Fecal Particles | 3.400 | 2.875 to 3.925 | Yes | **** | <0.0001 |
| 0:Without Fecal Particles vs. 72:Without Fecal Particles | 3.243 | 2.718 to 3.768 | Yes | **** | <0.0001 |
| 2:With Fecal Particles vs. 2:Without Fecal Particles | -1.037 | -1.562 to -0.5116 | Yes | **** | <0.0001 |
| 2:With Fecal Particles vs. 5:With Fecal Particles | -0.1633 | -0.6884 to 0.3617 | No | ns | 0.9788 |
| 2:With Fecal Particles vs. 5:Without Fecal Particles | -0.4233 | -0.9484 to 0.1017 | No | ns | 0.1835 |
| 2:With Fecal Particles vs. 24:With Fecal Particles | -0.2433 | -0.7684 to 0.2817 | No | ns | 0.8136 |
| 2:With Fecal Particles vs. 24:Without Fecal Particles | -0.1933 | -0.7184 to 0.3317 | No | ns | 0.9413 |
| 2:With Fecal Particles vs. 72:With Fecal Particles | 0.1800 | -0.3451 to 0.7051 | No | ns | 0.9613 |
| 2:With Fecal Particles vs. 72:Without Fecal Particles | 0.02333 | -0.5017 to 0.5484 | No | ns | >0.9999 |
| 2:Without Fecal Particles vs. 5:With Fecal Particles | 0.8733 | 0.3483 to 1.398 | Yes | *** | 0.0003 |
| 2:Without Fecal Particles vs. 5:Without Fecal Particles | 0.6133 | 0.08826 to 1.138 | Yes | * | 0.0144 |
| 2:Without Fecal Particles vs. 24:With Fecal Particles | 0.7933 | 0.2683 to 1.318 | Yes | ** | 0.0010 |
| 2:Without Fecal Particles vs. 24:Without Fecal Particles | 0.8433 | 0.3183 to 1.368 | Yes | *** | 0.0005 |
| 2:Without Fecal Particles vs. 72:With Fecal Particles | 1.217 | 0.6916 to 1.742 | Yes | **** | <0.0001 |
| 2:Without Fecal Particles vs. 72:Without Fecal Particles | 1.060 | 0.5349 to 1.585 | Yes | **** | <0.0001 |
| 5:With Fecal Particles vs. 5:Without Fecal Particles | -0.2600 | -0.7851 to 0.2651 | No | ns | 0.7544 |
| 5:With Fecal Particles vs. 24:With Fecal Particles | -0.08000 | -0.6051 to 0.4451 | No | ns | >0.9999 |
| 5:With Fecal Particles vs. 24:Without Fecal Particles | -0.03000 | -0.5551 to 0.4951 | No | ns | >0.9999 |
| 5:With Fecal Particles vs. 72:With Fecal Particles | 0.3433 | -0.1817 to 0.8684 | No | ns | 0.4208 |
| 5:With Fecal Particles vs. 72:Without Fecal Particles | 0.1867 | -0.3384 to 0.7117 | No | ns | 0.9520 |
| 5:Without Fecal Particles vs. 24:With Fecal Particles | 0.1800 | -0.3451 to 0.7051 | No | ns | 0.9613 |
| 5:Without Fecal Particles vs. 24:Without Fecal Particles | 0.2300 | -0.2951 to 0.7551 | No | ns | 0.8556 |
| 5:Without Fecal Particles vs. 72:With Fecal Particles | 0.6033 | 0.07826 to 1.128 | Yes | * | 0.0166 |
| 5:Without Fecal Particles vs. 72:Without Fecal Particles | 0.4467 | -0.07841 to 0.9717 | No | ns | 0.1388 |
| 24:With Fecal Particles vs. 24:Without Fecal Particles | 0.05000 | -0.4751 to 0.5751 | No | ns | >0.9999 |
| 24:With Fecal Particles vs. 72:With Fecal Particles | 0.4233 | -0.1017 to 0.9484 | No | ns | 0.1835 |
| 24:With Fecal Particles vs. 72:Without Fecal Particles | 0.2667 | -0.2584 to 0.7917 | No | ns | 0.7291 |
| 24:Without Fecal Particles vs. 72:With Fecal Particles | 0.3733 | -0.1517 to 0.8984 | No | ns | 0.3164 |
| 24:Without Fecal Particles vs. 72:Without Fecal Particles | 0.2167 | -0.3084 to 0.7417 | No | ns | 0.8921 |
| 72:With Fecal Particles vs. 72:Without Fecal Particles | -0.1567 | -0.6817 to 0.3684 | No | ns | 0.9839 |
