# Supplementary material for: Persistence of human enteric viruses in artificial and human saliva
Source: PLoS One. 2025 Dec 26;20(12):e0339724. doi: 10.1371/journal.pone.0339724 (PMC12742735; doi:10.1371/journal.pone.0339724)
Supplement: S8 Table — (DOCX) [file pone.0339724.s009.docx]

**Table S8:** Statistical comparisons for Figure 2A.

| **Tukey's multiple comparisons test** | **Mean Diff.** | **95.00% CI of diff.** | **Below threshold?** | **Summary** | **Adjusted P Value** |
| --- | --- | --- | --- | --- | --- |
| PBS vs. Saliva (Virus alone) | 15.41 | 9.785 to 21.04 | Yes | *** | 0.0003 |
| PBS vs. Saliva and fecal particles | 15.12 | 8.959 to 21.29 | Yes | *** | 0.0006 |
| PBS vs. Saliva (Virus + Oral Bacteria) | 15.41 | 9.785 to 21.04 | Yes | *** | 0.0003 |
| Saliva (Virus alone) vs. Saliva and fecal particles | -0.2890 | -5.915 to 5.337 | No | ns | 0.9978 |
| Saliva (Virus alone) vs. Saliva (Virus + Oral Bacteria) | 0.000 | -5.032 to 5.032 | No | ns | >0.9999 |
| Saliva and fecal particles vs. Saliva (Virus + Oral Bacteria) | 0.2890 | -5.337 to 5.915 | No | ns | 0.9978 |
