# Supplementary material for: Persistence of human enteric viruses in artificial and human saliva
Source: PLoS One. 2025 Dec 26;20(12):e0339724. doi: 10.1371/journal.pone.0339724 (PMC12742735; doi:10.1371/journal.pone.0339724)
Supplement: S9 Table — (DOCX) [file pone.0339724.s010.docx]

**Table S9:** Statistical comparisons for Figure 2C.

| **Uncorrected Fisher's LSD** | **Mean Diff.** | **95.00% CI of diff.** | **Below threshold?** | **Summary** | **Individual P Value** |
| --- | --- | --- | --- | --- | --- |
| PBS vs. *B. subtilis* | -30.80 | -53.43 to -8.173 | Yes | ** | 0.0098 |
| PBS vs. *S. downei* | -45.60 | -66.25 to -24.95 | Yes | *** | 0.0001 |
| PBS vs. *S. mutans* | -22.52 | -45.14 to 0.1088 | No | ns | 0.0510 |
| *B. subtilis* vs. *S. downei* | -14.80 | -35.46 to 5.854 | No | ns | 0.1518 |
| *B. subtilis* vs. *S. mutans* | 8.282 | -14.34 to 30.91 | No | ns | 0.4566 |
| *S. downei* vs. *S. mutans* | 23.08 | 2.428 to 43.74 | Yes | * | 0.0301 |
