# Supplementary material for: Persistence of human enteric viruses in artificial and human saliva
Source: PLoS One. 2025 Dec 26;20(12):e0339724. doi: 10.1371/journal.pone.0339724 (PMC12742735; doi:10.1371/journal.pone.0339724)
Supplement: S10 Table — (DOCX) [file pone.0339724.s011.docx]

**Table S10**: Data supporting figures 1-4

| **PBS** | ***B. subtilis*** | | ***S. downei*** | | | ***S. mutans*** | | | **PBS** | | | **Oral Bacteria** | |  |
| --- | --- | --- | --- | --- | --- | --- | --- | --- | --- | --- | --- | --- | --- | --- |
| 0.60 | 35.89 | | 19.33 | | | 6.90 | | | 10.23 | | | 51.14 | |  |
| 1.04 | 22.09 | | 44.17 | | | 16.56 | | | 8.18 | | | 20.45 | |  |
| 0.86 | 44.17 | | 88.34 | | | 15.18 | | | 8.18 | | | 32.73 | |  |
| 1.04 | 27.61 | | 20.71 | | | 38.65 | | | 4.09 | | | 46.02 | |  |
| 0.43 | 48.31 | | 33.13 | | | 17.94 | | | 12.27 | | | 12.27 | |  |
| 0.35 | 11.04 | | 66.26 | | | 44.17 | | | 16.36 | | | 50.11 | |  |
|  | **PBS** | | **Saliva (Virus alone)** | | | **Saliva (Virus + Fecal Particles)** | | | **Saliva (Virus + Oral Bacteria)** | | |  | |  |
|  | 12.33 | | ND | | | 0.19 | | | ND | | |  | |  |
|  | 18.49 | | ND | | | 0.39 | | | ND | | |  | |  |
|  | 15.41 | | ND | | | 0.29 | | | ND | | |  | |  |
| CVB3 | |  | |  |  | |  |  |  |  |  | |  | |
| **Hours** | | **PBS (PFU/mL)** | | | | | **Artificial Saliva (PFU/mL)** | | | **Human Saliva (PFU/mL)** | | | | |
| 0 | | 4.74 | | 4.96 | 5.28 | | 4.74 | 4.96 | 5.28 | 4.65 | 4.75 | | 4.67 | |
| 6 | | 4.31 | | 4.58 | 4.77 | | 3.95 | 4.21 | 4.61 | 3.20 | 2.60 | | ND | |
| 12 | | 2.78 | | 2.93 | 3.14 | | 2.30 | 2.90 | ND | 2.00 | 2.30 | | ND | |
| 24 | | 2.92 | | 2.89 | 2.84 | | 2.15 | 2.48 | 2.60 | ND | ND | | ND | |
| 48 | | 2.60 | | 2.48 | ND | | ND | ND | ND | ND | ND | | ND | |
| CVB3 | |  | |  |  | |  |  |  |  |  | |  | |
| **Hours** | | **With Particles (PFU/mL)** | | | | | **Without (PFU/mL)** | | |  |  | |  | |
| 0 | | 4.74 | | 4.96 | 5.28 | | 4.74 | 4.96 | 5.28 |  |  | |  | |
| 6 | | 4.18 | | 4.48 | 4.71 | | 3.95 | 4.21 | 4.61 |  |  | |  | |
| 12 | | 3.35 | | 3.68 | 3.81 | | 2.30 | 2.90 | ND |  |  | |  | |
| 24 | | 2.39 | | 2.54 | 2.60 | | 2.15 | 2.48 | 2.60 |  |  | |  | |
| 48 | | 2.00 | | 2.30 | ND | | ND | ND | ND |  |  | |  | |
| CVB3 | |  | |  |  | |  |  |  |  |  | |  | |
| **Hours** | | **With Particles (PFU/mL)** | | | | | **Without (PFU/mL)** | | |  |  | |  | |
| 0 | | 4.65 | | 4.75 | 4.67 | | 4.65 | 4.75 | 4.67 |  |  | |  | |
| 6 | | 2.90 | | 3.20 | 2.60 | | 3.20 | 2.60 | ND |  |  | |  | |
| 12 | | 2.59 | | 2.84 | 2.60 | | 2.00 | 2.30 | ND |  |  | |  | |
| 24 | | 2.00 | | 2.30 | ND | | ND | ND | ND |  |  | |  | |
| 48 | | ND | | ND | ND | | ND | ND | ND |  |  | |  | |
| AdV41 | |  | |  |  | |  |  |  |  |  | |  | |
| **Hours** | | **PBS (IU/mL)** | | | | | **Artificial Saliva (IU/mL)** | | | **Human Saliva (IU/mL)** | | | | |
| 0 | | 5.60 | | 5.60 | 5.81 | | 5.60 | 5.60 | 5.81 | 6.29 | 6.29 | | 6.29 | |
| 6 | | 5.18 | | 5.65 | 5.58 | | 6.01 | 6.25 | 6.08 | 3.57 | 4.50 | | 4.25 | |
| 12 | | 5.55 | | 5.51 | 5.47 | | 6.01 | 6.05 | 6.20 | 3.44 | 3.26 | | 3.78 | |
| 24 | | 5.52 | | 5.25 | 4.60 | | 4.71 | 5.47 | 5.66 | 3.23 | 3.30 | | 3.26 | |
| 72 | | 4.92 | | 4.78 | 5.18 | | 6.00 | 5.81 | 5.88 | 3.00 | 2.96 | | 3.18 | |
| AdV41 | |  | |  |  | |  |  |  |  |  | |  | |
| **Hours** | | **With Fecal Particles (IU/mL)** | | | | | **Without Fecal Particles (IU/mL)** | | |  |  | |  | |
| 0 | | 5.60 | | 5.60 | 5.81 | | 5.60 | 5.60 | 5.81 |  |  | |  | |
| 6 | | 5.60 | | 5.85 | 5.91 | | 6.01 | 6.25 | 6.08 |  |  | |  | |
| 12 | | 5.59 | | 5.43 | 5.49 | | 6.01 | 6.05 | 6.20 |  |  | |  | |
| 24 | | 5.01 | | 5.22 | 4.54 | | 4.71 | 5.47 | 5.66 |  |  | |  | |
| 72 | | 5.48 | | 5.62 | 5.45 | | 6.00 | 5.81 | 5.88 |  |  | |  | |
| ADV41 | |  | |  |  | |  |  |  |  |  | |  | |
| **Hours** | | **With Fecal Particles (IU/mL)** | | | | | **Without Fecal Particles (IU/mL)** | | |  |  | |  | |
| 0 | | 6.29 | | 6.29 | 6.29 | | 6.29 | 6.29 | 6.29 |  |  | |  | |
| 6 | | 3.02 | | 3.10 | 3.09 | | 3.57 | 4.50 | 4.25 |  |  | |  | |
| 12 | | 3.17 | | 3.27 | 3.26 | | 3.44 | 3.26 | 3.78 |  |  | |  | |
| 24 | | 3.21 | | 3.35 | 3.38 | | 3.23 | 3.30 | 3.26 |  |  | |  | |
| 72 | | 2.90 | | 2.90 | 2.87 | | 3.00 | 2.96 | 3.18 |  |  | |  | |
